# Design and development of highly conserved, HLA-promiscuous T cell multiepitope vaccines against human visceral leishmaniasis

**DOI:** 10.3389/fimmu.2025.1540537

**Published:** 2025-03-31

**Authors:** Aline Silva Barreto, Mariana Nobre Farias de Franca, Tatiana Leão dos Santos dos Reis, Joao Antonio Barbosa Martins Silva, Priscila Lima Dos Santos, Fabrícia Alvisi de Oliveira, Angela Maria da Silva, Lucas Sousa Magalhaes, Danielle Angst Secco, Maria Aiza Fontes Andrade, Luís Cristóvão Porto, Daniela Santoro Rosa, Rafael Ciro Marques Cavalcante, Cristiane Bani Corrêa, John Sidney, Alessandro Sette, Roque Pacheco de Almeida, Clarisa Beatriz Palatnik-de-Sousa

**Affiliations:** ^1^ Molecular Biology Laboratory, University Hospital, Department of Medicine Federal University of Sergipe, Aracaju, Sergipe, Brazil; ^2^ Postgraduate Program in Health Science, Federal University of Sergipe, Aracaju, SE, Brazil; ^3^ Department of Morphology, Federal University of Sergipe, Aracaju, Sergipe, Brazil; ^4^ Institute of Microbiology Paulo de Góes, Federal University of Rio de Janeiro (UFRJ), Rio de Janeiro, Brazil; ^5^ Postgraduate Program in Plant Biotechnology and Bioprocesses, Center for Health Sciences, Federal University of Rio de Janeiro, Rio de Janeiro, RJ, Brazil; ^6^ Department of Medicine, Federal University of Sergipe, Aracaju, Brazil; ^7^ TIXUS Technologic Core for Tissue Repair and Histocompatibility, Rio de Janeiro State University, Rio de Janeiro, Brazil; ^8^ Department of Pharmacy and Graduate program in Applied Heath Science, Federal University of Sergipe, Lagarto, Sergipe, Brazil; ^9^ Department of Microbiology, Immunology and Parasitology, Federal University of Sao Paulo, São Paulo, Brazil; ^10^ Division of Vaccine Discovery, La Jolla Institute for Immunology, La Jolla, CA, United States; ^11^ Division of Infectious Diseases and Global Public Health, Department of Medicine, University of California, San Diego, La Jolla, CA, United States

**Keywords:** multiepitope based vaccines, leishmaniosis, visceral leishmaniosis, HLA class I and class II promiscuous epitopes, *Leishmania* conserved antigens

## Abstract

**Introduction:**

No vaccine is currently licensed against human visceral leishmaniasis (VL), a fatal CD4+ T cell immunosupressive disease against which chemotherapy is reduced to a few toxic drugs. The NH36 nucleoside hydrolase is a DNA metabolism vital enzyme present in all *Leishmania* species. A vaccine based on such a conserved antigen could protect against both VL and cutaneous leishmaniasis, whose epidemics geographically overlap. Increased frequencies of NH36-specific IL-2+TNF-α+IFN-γ+-producing CD4+ T cells were associated with VL immune protection.

**Methods:**

the sequences of HLA-Class I and Class II T cell epitopes were predicted in the NH36 peptide sequence using the Tepitope, Propred, IEDB and NetMHCpan EL 4.1 immune informatic tools. The epitopes were synthetized and used to study their reactivity with sera samples, and to stimulate the *in vitro* response of PBMC of human patients cured from VL, asymptomatic individuals and healthy blood donors of a non-endemic area. Cytokine production was studied intracellularly by flow cytometry (ICS) and cytokine secretion was measured in PBMC supernatants. The HLA typing of DNA patients and the analysis of epitope conservancy in the *Leishmania* genus were obtained. Two recombinant multiepitope proteins were designed, cloned in *E. coli*, expressed, purified and used for *in vitro* stimulation of PBMC of VL cured and asymptomatic patients.

**Results:**

We identified *in silico* fifteen NH36 conserved epitopes that correspond to promiscuous binders of HLA-DR, -DQ, -DP class II molecules, as well as HLA-A, B and C class I molecules. Collectively, these epitopes provide high worldwide population coverage of both class I and II alleles, and bound to alleles associated with VL susceptibility and resistance. VL asymptomatic individuals showed maximal frequencies of CD4+ and CD8+ multifunctional IL-2+TNF-α+IFN-γ+-producing T lymphocytes in response to these epitopes, with secretion of TNF-α, IL-1β and IL-6. Two recombinant multiepitope vaccines were designed using these epitopes linked by AAA or GPGPG spacers. Both proteins promoted CD4+ and CD8+ T cell responses in PBMC of VL cured and asymptomatic individuals.

**Discussion:**

Both MultiAAA and MultiGPGPG proteins could be potentially used for universal human vaccination against leishmaniasis.

## Introduction

Human visceral leishmaniasis (VL), the world 9th highest incidence vector-borne disease is fatal if not treated soon after symptoms begin (1). VL is solely anthroponotic in the Indian Subcontinent (ISC) and Eastern Africa, and a canid zoonosis in the Mediterranean basin, China, the Middle East and South America (2, 3). It shows increasing lethality rates and relapse in East Africa and Brazil (4–6), due to global warming (7), other parasitic co-infections (8–10) and among HIV co-infection individuals (1, 2).

Clinical signs of VL include fever, malaise, enlargement of spleen and liver, anemia, leukopenia, weight loss, hypergammaglobulinemia and progressive suppression of the CD4^+^ T cell response, with decreased numbers of CD4^+^ total counts and *Leishmania*-specific CD4^+^ T cells (6, 11). For these reasons, it has been proposed that vaccine antigens that could stimulate CD4^+^ Th1 T cell responses may generate effective protection against VL (3, 12, 13), while CD8^+^ cytotoxic responses might contribute as well to VL prevention and therapeutic (12, 14).

Vaccines are the best tools for infectious disease prevention (15). The history of the development of vaccines against leishmaniasis has been reviewed elsewhere (16–18). There is no currently licensed vaccine against human VL and its chemotherapy is highly toxic (16). We developed the first licensed canine VL vaccine, Leishmune ^®^, of high prophylactic efficacy and safety (19–22), which blocks transmission (23, 24) reduces canine and human cases of VL (25) and promotes the clinical, parasitological and sterile cure of infected dogs (26, 27). Its main antigen is the Nucleoside hydrolase NH36 of *Leishmania (L.) donovani*, a DNA metabolism enzyme (28) which is a conserved marker of the *Leishmania* genus (29). A vaccine based on a conserved *Leishmania* antigen, such as NH36, could control both epidemics of VL and cutaneous leishmaniasis (CL) that geographically overlap (23, 30–36).

The role of TNF-α in VL has been shown to be ambivalent. It is active on the outcome of infection but also on its cure or generalization. In addition, TNF-α, IL-1β and IL-6 were considered important in both the induction and effector phases of the immune responses (37). In fact, circulating serum levels of TNF-α were ten-fold increased, while IL-1β levels were low in VL patients and serum TNF-α levels were also inversely related to IL-1β release *in vitro* (37). In contrast, increased serum levels of TNF, IL-6 and IL-1β were associated to inflammation and VL pathogenesis and reversed post initiation of leishmanicidal treatment (38). Levels of IL-1β (39), IL-6 (39–43), and TNF-α (40, 43–45) were increased during active disease and are considered key cytokines associated with inflammation, also previously linked to VL pathogenesis (38). In addition, high levels of TNF-α, IL-1β, IL-6 have been independently implicated with disease severity and death associated with VL (39, 43). Mostly, IL-6 is the central cytokine associated with lethal disease and its inhibition is a potential target of adjuvant therapy for severe or pediatric forms of VL (39). Enhanced systemic IL-6 production was also found in sera from dogs with active VL while, in contrast, TNF-alpha did not differ between VL patients and heathy controls and IL-6 has been suggested as a good marker of active disease during leishmaniasis (46).

In contrast, confirming the ambivalent role of TNF alfa in VL, it has also been showed to be involved in protection against the disease, by activating and inducing NO macrophage production to kill amastigotes, inducing granulomas and wound healing process and by promoting IL-10 T cell production for immune homeostasis (47). In addition, a Th1 chimeric protein determined a remarkable inhibition of splenic parasitic multiplication positively correlated with boosted Th1 dominant immune response against lethal *L. donovani* challenge in hamsters as evidenced by high IFN-*γ* and TNF-*α* and low IL-10 (48). In mice vaccinated with the NH36 domains, increases in DTH and in ratios of TNFα/IL-10 CD4^+^ producing cells were strong correlates of protection confirmed by a pronounced and long-lasting decrease in parasite load (30).

Recently, confirming the strong immunogenicity of the NH36, we assessed in active and cured patients and VL asymptomatic subjects the clinical signs and cytokine responses to the *Leishmania donovani*-nucleoside hydrolase NH36 antigen and its N-(F1), central (F2) and C-terminal (F3) domains. As markers of VL resistance, the F2 induced the highest levels of IFN-γ, IL-1β and TNF-α and, together with F1, the strongest secretion of IL-17, IL-6 and IL-10 in DTH+ asymptomatic and cured subjects (12). Proliferation in response to NH36 and F1 was also accompanied by a significant increase of IFN-γ and TNF-α secretion in cured VL patients of Spain (49). Additionally, we identified that the Th1 and Th17 CD4^+^ response of PBMC from cured or asymptomatic Delayed hypersensitivity positive (DTH^+^) VL human patients is directed to the F1 (12, 49) and F2 domains while the CD8^+^ response of untreated patients is directed to the F3 and F1 domains of NH36 (12). These results indicated that the whole sequence of NH36 is immunogenic and that it holds epitopes that could generate protection in both susceptible and resistant individuals.

We and others demonstrated previously that mapping and using for vaccination, the most immunogenic domains of a protein can optimize vaccine efficacy over the use of the full length protein (30, 33, 36, 50, 51). The combination of the main immunogenic domains into chimeras expressed *in tandem* promoted higher vaccine efficacy than the use of the individual domains or their simple mixture (34, 35). In this study, we chose to use multiepitope vaccines rather than the full-length protein for safety and immune efficacy optimization reasons.

Recently, the concept that the most effective immune response against pathogens is derived from several different T cells that respond to a set of short pathogen-derived peptide epitopes has become prevalent (52). Thus, epitope mapping is one of the strategies that can be used for the design of safe multiepitope vaccines. In this way, a single universal vaccine can bring together the most dominant epitopes of a protein, or of different proteins from a strain, or of conserved proteins from different strains of a species or genus (52, 53). Nonetheless, the development of such vaccines might be challenging, unless the extensive polymorphism in human leukocyte antigens (HLA) is taken into consideration.

Our approach aims to develop multiepitope universal vaccines against VL based on the highly conserved NH36 antigen. This will be done by selecting its most promiscuous and conserved epitopes, which provide the highest HLA population coverage worldwide. These vaccines could be used alone, or in combination with other *Leishmania* protein vaccines, in order to prevent and control VL.

## Material and methods

### *In silico* predictions of HLA-class II and class I epitopes of nucleoside hydrolase (NH36)

Immunoinformatic predictions were obtained on the complete sequence of the *Leishmania donovani* nucleoside hydrolase NH36 (EMBL, Genbank and DDJB data bases: AY007193 and AAG02281.1 access codes; SWISS-PROT: Q8WQX2 UNi-Prot access code) to identify the potentially promiscuous epitopes which bind most class II histocompatibility molecules. Predictions were run using the IEDB recommended 2.22 method (http://tools.iedb.org/mhcii/), (PR < 5%, 10% and 20%), for the 27 most frequent alleles of the human HLA class II molecules (DRB1, DRB3, DRB4, DRB5, DQA1/DQB1 and DPA1/DPB1), the PROPRED (DRB1, PR < 3%) and the TEPITOPE (DRB1, PR < 3%) tools. Initially, 12 epitopes (1-2014, 2-1014, 3-2014, 1-2018, 3-2018, 5-2018, 7-2018, 9-2018, 11-2018, 13-2018, 15-2018 and 17-2018) were identified, synthesized by Genscript and studied for their reactivity with serum antibodies, on intracellular cytokine production by CD4^+^ and CD8^+^ T cells, and on cytokine secretion in PBMC supernatants from asymptomatic infected and VL cured human patients. Although the search was focused on 20 peptide length epitopes, some minor differences were detected in the results obtained with the three algorithms. Therefore, while 9 among the 12 initial epitopes are composed of 20 amino acids (**Supplementary Table S2**), epitopes 1-2018 and 11-2018 show 21 amino acids and epitope 13-2018 contains 24 amino acids. Furthermore, as the epitopes 15-2018 and 17-2018 had a high overlap of 16 amino acids, they were condensed into the new epitope 15-17-2018 (24 amino acids). IEDB tool also retrieved the additional BL1 (18 amino acids) and BL2 epitopes (20 amino acids) (**Supplementary Table S2**). This amino acid range was chosen to balance the inclusion of flanking regions with practical considerations for synthesis and experimental testing. Binding capacity was also assessed using *in vitro* assays based on the epitope ability to inhibit the binding of a radioactive peptide probe to purified HLA molecules (54). The affinity of NH36 epitopes for the HLA-DRB1 (55, 56) and DQB1 (56) alleles associated with resistant/protective, high risk or intermediate-risk of contracting VL was also determined. For *in vitro* assays, IC50 concentrations ≤ to 1000 ηM was utilized as a threshold to define binding (57). Additionally, HLA class I binding predictions for HLA-A and -B were determined for the 15 class II epitopes using the NetMHCpan EL 4.1 software, which scores 9- and 10-mers (IEDB Recommended 2020.09; http://tools.iedb.org/mhci/). The Net-MHC 4.0 tool (https://services.healthtech.dtu.dk/services/NetMHC-4.0/) was used for HLA-C prediction. Binding was defined using the ≤1% threshold (58). The number of possible ligands for 27 common HLA-A, -B and the 10 common HLA-C class I molecules was tallied for each epitope.

### NH36 epitopes population coverage and affinity to HLA-alleles associated to VL susceptibility or resistance

The World population coverage of the set of 15 predicted sequences was disclosed using the IEDB platform (http://tools.iedb.org/population/). Population coverage as determined by the tool considers only DRB1, DQB1 and DPB1 specificities, and thus may be an underestimate.

### Patients

Patients diagnosed with VL, based on fever, weight loss, anemia, enlarged spleen or liver (measured in centimeters, below the ribs’ lower edge), hypergammaglobulinemy, pancytopenia, positive serum reactivity in the KalazarDetect^®^ Rapid Test (INBIOS International Inc., Seattle, WA) and positive culture in NNN media (Sigma-Aldrich), were treated at the UFS University Hospital, SE, Brazil. PBMC of blood samples were collected from VL cured patients (n=10), family members or contacts of patients who were positive for the DTH test with *Leishmania* antigen (Montenegro test) (n=10), and non-endemic area healthy controls (n=10) and incubated with NH36 synthetic epitopes. We considered infected asymptomatic patients, the family members and contacts of patients who underwent DTH+ test and presented a papule measuring 5 mm or larger on the forearm. Cured patients were the individuals who completed VL treatment with no recurrence within 180 days. Cured or VL patients aged 12-50. Exclusion criteria included pregnant women, alcoholic patients, children, co-infection with HIV, Hepatitis and other pathologies of infectious origin. Samples of healthy blood donors obtained from the Clementino Fraga Filho-UFRJ University Hospital Blood Bank showed serum negative responses in tests for HIV, Chagas disease, Hepatitis B and C, Syphilis, HTLV and Chagas Disease. Buffy coats from these controls used for intracellular staining (ICS) tests were also obtained, thanks to the project approved for Dr P.R. Zuquim Antas of the Oswaldo Cruz Institute of Rio de Janeiro (CAAE 35775014.0.0000.5248). Furthermore, PBMC from VL cured patients (n = 4), DTH+ (n = 3) and endemic area healthy blood donor controls from Sergipe Hemotherapy Center (n = 4) were incubated with the MultiAAA and the MultiGPGPG proteins. This study followed the guidelines and regulations of the Brazilian National Council of Health resolution 196/96 (CAAE 0162.0.107.000-09) approved by the Federal University of Sergipe Research Ethics Committee. The objectives of the study were explained to all invited participants who gave written informed consent in accordance to the Declaration of Helsinki.

### Antibody responses to NH36 synthetic epitopes

Plates were sensitized with 50 µg of each synthetic peptide (1-2014, 2-2014, 3-2014, 1-2018, 3-2018, 5-2018, 7-2018, 9-2018, 11-2018, 13-2018, 15-2018 and 17-2018) diluted in MilliQ and blocked with 2% BSA supplemented PBS buffer. Sera samples were diluted (1:100), plated, incubated 1h at 37°C, followed by 5 PBS* buffer washes (0.018 M PBS pH 7.2; 1% skimmed milk, 0.05% Tween 20), then added 100 µl protein A peroxidase conjugates (Kirkegaard & Perry Laboratories, USA) diluted 1:1000, anti-human IgG1-peroxidase (Life Technologies, USA), anti-human IgG2-peroxidase (Invitrogen, USA), anti-human IgG3-peroxidase (Invitrogen), or anti-human IgG4-peroxidase IgG4 (Life technologies) diluted 1: 2000. Plates were incubated at 37°C for 1 hour, washed 5 times with PBS^*^ buffer and developed with 100 μl/well of Sigma^®^ Ortho Phenylene Diamine (OPD) solution in buffer pH 5.2. Reactions were stopped with 1 N sulfuric acid and absorbances were read in an ELISA reader (BioRad) at 492 ηm.

### Flow cytometry T cell assays

The intracellular staining (ICS) (59) and the activation-induced marker (AIM) methods (60) were described in detail before. PBMCs were isolated using Ficoll-Hypaque density gradient (Pharmacia AB, Upsalla, Sweden), platted (10^6^/well), incubated for 6 hours at 37 °C and 5% CO_2_ with 25 µg/ml of each synthetic epitope (1-2014, 2-2014, 3-2014, 1-2018, 3-2018, 5-2018, 7-2018, 9-2018, 11-2018, 13-2018, 15-2018 and 17-2018) or 10 µg/ml SLA. In another set of experiments, PBMC were incubated with 12.5 µg/ml of MultiAAA or MultiGPGPG proteins 12.5 µg/ml of the mixture of 13 synthetic epitopes, which compose MultiAAA and MultiGPGPG proteins (0.96 ug/ml of each epitope: 1-2014, 2-2014, 3-2014, 1-2018, 3-2018, 5-2018, 7-2018, 9-2018, 11-2018, 13-2018, 15-17-2018, BL1-2018 and BL2-2018 or with 10 µg/ml SLA or with no addition as controls. The epitope 15 and 17-2018 were overlapping, therefore were resumed into the new epitope 15-17-2018. Epitopes BL1 and BL2 were retrieved using the IEDB tool at PR10% and 20%. All mixtures were cultured for additional 12 h with 1 ug/ml Brefeldin (Golgi Plus BD Biosciences). For ICS, cells were labeled with the anti-human CD3 Pecy7 (HIT3a), CD45 APCcy7 (HI100), CCR7 PE (G043H7) (Biolegend^®^, CA, USA) and CD4 V500 (RPA-T4), CD8 Pecy5 (RPA-T4) (BD Bioscience, NJ, USA) antibodies, washed and permeated with 50µl of Citofix/Cytoperm^®^ kit (BD Bioscience^®^, NJ, USA), and further incubated with rat anti-human IL-2 BV421 (MQ1-17H12, BD Horizon ™), IFN-γ Alexa 488 (4S.B3, BioLegend ^®^), TNF APC (Mab 11, BD Bioscience ^®^), washed and analyzed using a FACS Canto II Flow Cytometer of BD with Diva 6.0 Software, for 30,000 events. Compensation and analysis was performed using the Boolean analysis of FlowJo Software 10.0 (61) based on any combination of IFN-γ, IL-2 or TNF-α (62). For the gate strategy initially, the viable cell population was separated in order to exclude the excess of “debris” and dead cells. Within this population the lymphocytes were selected using the parameters size by granularity (FSC x SSC) (**Supplementary Figure S1**). Only those lymphocytes that were CD3^+^ and later, those that were CD3^+^CD4^+^ and CD3^+^CD8^+^ were selected. The population of naïve lymphocytes was excluded (CCR7^+^CD45^+^) in order to evaluate only the CD3^+^CD4^+^ and CD3^+^CD8^+^ lymphocyte populations that became effector and memory lymphocytes, to reflect a better immune response (**Supplementary Figure S1**). For ICS experiments, the frequencies of antigen-stimulated lymphocytes of each patient were discounted from the background values of its PBMC incubated with no antigen addition.

For AIM, after antigen stimulation, cells were labeled with mouse anti-human CD3 Pecy7 (SK7), CD4 V500 (RPA-T4), CD8 PeCy5 (HIT8α) (BD Bioscience^®^, NJ, USA), CD69 BV421 (FN50), CD40L FITC (24–31) and CD137 Alexa 647 (4B4-1) (BioLegend^®^, CA, USA) antibodies, washed, and analyzed using a BD FACS Canto II Flow Cytometer with Diva 6.0 Software, for 30,000 events. Data analysis was performed using FlowJo Software 10.9.0. As described for ICS, among the CD3^+^ cells, CD4^+^ AIM cells were identified as CD69^+^ CD40L^+^ and the CD8^+^ AIM cells, were recognized as CD69^+^ CD137^+^ cells (**Supplementary Figure S2**). AIM results of each patient were expressed as stimulation indices (SI), calculated as the fold increase response to the antigens over its own background values, with no antigen addition.

### Cytokine response in PBMC supernatants

Secretion of IL-2, TNF-α, IFN-γ, IL-12p70, IL-1β, IL-4, IL-6, TGF-β, IL-22, IL-10 cytokines into supernatants of epitope-stimulated PBMC was evaluated in the DTH^+^ individuals using the Luminex system (12). A baseline control with cells from each DTH^+^ individual was used, without stimulus, as a comparison parameter.

### HLA typing of DNA patients

The HLA-DNA typing for 11 HLA *loci* alleles (HLA-A*, HLA-B*, HLA-C*, DRB1*, DRB3*, DRB4*, DRB5*, DQA1*, DBQ1*, DPA1*, DPB1*) was determined for 8 sick patients with VL, after cure, and 10 DTH^+^ subjects. Blood samples were collected in tubes containing citric acid, sodium citrate and dextrose and underwent erythrocyte lysis using a 1.6 M Sucrose, 5% v/v Triton X-100, 25 mM Magnesium Chloride and 1 M Tris HCl pH 7.5 containing buffer. Subsequent DNA extraction from the nucleated cells was carried out using the PureLink^®^ Genomic DNA Kit^®^ (Thermo-Fisher Sci., Waltham, MA, USA). Concentration and purity of the extracted DNA were determined using a NanoDrop Lite Spectrophotometer (Thermo-Fisher Sci). DNA samples were analyzed by Next Generation Sequencing (NGS) using the Holotype HLA Assay (Omixon Inc., Budapest, Hungary) and the MiSeq Reagent Nano v2 (Illumina, San Diego, USA), for HLA-A, -B, C-, DRB1, -DRB3/4/5, -DQA1, -DQB1, DPA1- and -DPB1, according to the manufacturer’s instructions. Allelic and haplotypic frequencies, as well as the Hardy-Weinberg balance test, were evaluated using Arlequin 3.5 software (63).

### Analysis of epitope conservancy

Identity of each NH36-epitope was compared with that of the epitopes of other *Leishmania* species NHs included in the Blastp database (https://blast.ncbi.nlm.nih.gov/Blast.cgi?PROGRAM=blastp&PAGE_TYPE=BlastSearch&LINK_LOC=blasthome). For each epitope, Blastp returned the percent identity to 100 different proteins from which were excluded those that did not belong to the genus *Leishmania* and the hypothetical proteins, leaving 35 proteins. The number of genomes retrieved for each species was variable: 16 genomes of *Leishmania (L.) donovani*, followed by 6 of *Leishmania (L.) infantum*, 1 of *Leishmania (L.) infantum chagasi*, 1 of *Leishmania (L.) tropica*, 5 of *Leishmania (L.) major*, 2 of *Leishmania (L.) mexicana*, 1 of *Leishmania (L.) tarentolae*, 1 of *Leishmania (V.) panamensis*, 3 of *Leishmania (V.) braziliensis* and 2 of *Leishmania (V.) guyanensis*.

### Multiepitope proteins design, physicochemical properties, allergenicity and toxicity predictions

The MultiAAA and MultiGPGPG proteins were planned using the NH36 HLA-class II epitopes linked by three Alanine (64) or by Glycine-Proline-Glycine-Proline-Glycine (65–67) spacers, respectively, to avoid the formation of neoepitopes. The molecular weight (MW), theoretical isoelectric point (pI), amino acid composition, *in vitro* and *in vivo* half-life, aliphatic index, instability and grand average of hydropathicity (GRAVY) physicochemical properties were predicted with the Expasy ProtParam tool (https://web.expasy.org/protparam/). Toxicity was predicted using the Toxin pred2 (ML, Merci, Blast, Hybrid scores)(https://webs.iiitd.edu.in/raghava/toxinpred2/batch.html) and the PSI-BLAST BTXpred tools (https://webs.iiitd.edu.in/raghava/toxinpred/) and allergenicity, using the AllerTOPv.2.0 tool (https://www.ddg-pharmfac.net/AllerTOP/).

### Prediction of secondary structure and modeling of tertiary structure

Secondary structures predictions were run using the PSIPRED tool (http://bioinf.cs.ucl.ac.uk/psipred/) which also predicts the transmembrane topology, helix, strand, coil and domain recognition. Tertiary structures molecular modelling was obtained using the Swiss-Model tool (https://swissmodel.expasy.org/) by homology with the *Leishmania major* NH crystal structure pdb (1EZR) (https://www.rcsb.org) and PyMOL 2.5.7 program for graphic presentation. Refinement of the models run using the Swiss Model and the PROCHEK-PDBSUM tool (http://www.ebi.ac.uk/thornton-srv/databases/cgi-bin/pdbsum/GetPage.pl?pdbcode=index.html). For model validation, the Ramachandran plots, their best Q mean Z score, the highest Qmean Disco Global and the highest GMQE disclosed by the Swiss Model, and the highest overall G factor, obtained by the PROCHEK-PDBSUM tool were assessed. Qmean of the Swiss Model tool is an estimator known as z-score. When the value is close to 0, the model is reliable and, therefore, there is a good agreement between the model and the experimental structures. The G factor gives an estimate of how unusual the model is (bellow -0.5 = unusual; bellow -1 = highly unusual).

### Codon optimization, cloning, expression and purification

SnapGene (version 6.0.2) (https://www.snapgene.com/) was used to integrate the adapted DNA sequence to pET-28b (+) vector. NetNGlyc - 1.0 was used for determination of the N-glycosylation sites in human protein (https://services.healthtech.dtu.dk/service.php?NetNGlyc-1.0). *Escherichia coli* (strain K12) was selected as the target organism and the two multiepitope proteins were cloned with optimized codons for *E. coli* into the pET28b (+) between the NcoI and XhoI restriction sites, with one 6 His tag at its C-terminal end (Genscript, NJ, USA). *E. coli* BL21 (DE3) cells were transformed with the MultiAAA-pET28b or MultiGPGPG-pET20b plasmids and induced for expression using 0.5 mM, 0.75 or 1 mM IPTG or with no addition for 2 hours at 37°C, 200 rpm and further centrifuged at 10,000 rpm for 5 min. at 4°C. Pellets were lysed in lysis buffer (Tris-HCl 20 mM, NaCl 500 mM, Lysozyme 1 mg/mL, PMSF 1 mM, pH 8) using an Ultra-Turrax^®^ Tube Drive Ika dispenser. The recombinant proteins soluble and insoluble fractions were separated by centrifugation and analyzed using 12% SDS-PAGE under denaturing conditions. Pellets obtained after bacterial lysis were washed twice using inclusion body washing buffer (20 mM Tris-HCl, 500 mM NaCl, 10% Glycerol, 4 M Urea, 0.5% CHAPS, pH 7) and centrifuged at 10,000 rpm for 15 minutes at 4°C. Pellet was resuspended in solubilization buffer (20 mM Tris-HCl, 500 mM NaCl, 10% Glycerol, 8 M Urea, pH 7), kept under agitation overnight at 17°C to ensure complete solubilization of the inclusion bodies and centrifuged at 10,000 rpm for 60 minutes at 4°C to remove any insoluble residue. The recombinant proteins were then refolded and purified by affinity chromatography in Ni Sepharose Excel HisTrap column (Cytiva™,MA, USA). The refolding process was carried out on the column using a decreasing urea gradient (8 M to 0 M). The renatured proteins were eluted with 120 mM imidazole, analyzed by SDS-PAGE, concentrated and desalted with PBS using the Ultra-4 Amicon device^®^ with 10 kDa ultrafiltration membranes and assayed for protein content. Purity of the protein preparations along the purification process was monitored by SDS-PAGE (**Supplementary Figure S3**). The absence of LPS was confirmed using the Chromogenic Endotoxin Quant Kit, Pierce™ (ThermoScientific). The United States Pharmacopeia considers endotoxin concentrations up to 5 UE/mL/Kg/h as acceptable (68) and all preparations showed concentrations below 0.1 EU/mL (endotoxic unities per ml). The frequencies of CD3^+^CD4^+^ lymphocytes were similar in all groups of individuals.

### Prediction of the immune stimulations response to the multiepitope vaccine

To this aim the C-ImmSim software was used (https://kraken.iac.rm.cnr.it/C-IMMSIM/) (69, 70) considering the administration of three vaccine doses, with 28 days interval and no adjuvant, using 1,000 epitopes vaccine per dose. The period analyzed included 365 days. A sequence of 46 Alanine residues was used as a negative control and the sequence of the Full=Probable citrate synthase, mitochondrial, Flags: Precursor UniProtKB/Swiss-Prot: A4H9H8.1, as a positive control.

### Statistical analysis

The ANOVA method with the Sidak´s multicomparison test was used for comparison of sera absorbance values and 95% Confidence interval (95% CI) was used to compare the AIM, total frequencies and frequencies of CD4^+^ and CD8^+^ T cells producing one, two or three cytokines and the concentrations of cytokines secreted to PBMC supernatants. The percents of CD3^+^CD8^+^ T cells were slightly higher for DTH^+^ subjects. The *x* ^2^ test with Yates correction was used to compare the frequencies of each HLA-class I or class II alleles of VL cured and asymptomatic DTH^+^ patients (scipy.stats chy.square contingency library of Phyton).

## Results

### Epitope prediction, promiscuity and affinity for HLA DR, DQ and DP molecules *in silico* and *in vitro*

**Figure 1:**
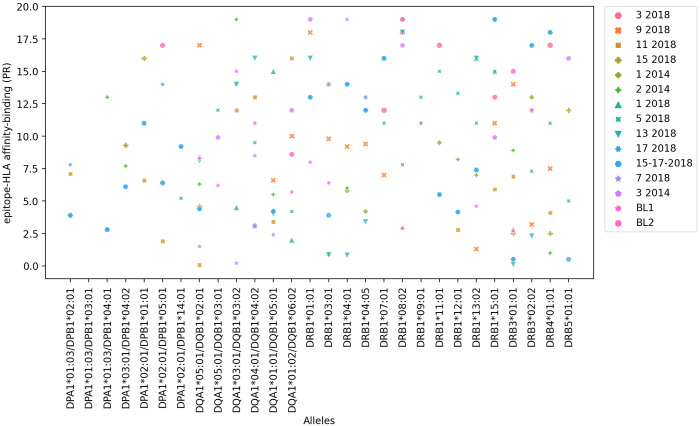
*In silico* epitope to HLA allele affinity-binding prediction. NH36 epitope affinity-prediction for the 27 most frequent HLA-class II human alleles of the DRB1*, DRB3*, DRB4*, DRB5*, DQA1*/DQB1* and DPA1*/DPB1* genes was obtained using the IEDB HLA-class II tool. The *y* axis shows the Percentile Rank (PR) with which each epitope binds to and HLA-molecule. The lowest the percentile rank (PR) the strongest the affinity of the epitope and HLA-molecule interaction. PR < 10% and PR < 20% values were considered for a restricted and more broader analysis, respectively (**Supplementary Table S1**).

Candidate epitopes were selected using a combination of bioinformatics and immunochemical approaches, as described in the Methods section, that assessed epitope capacity to bind a panel of 27 most common HLA-DRB1, -DRB3, -DRB4, -DRB5, DQA1/DQB1 (DQ) and DPA1/DPB1 (DP) class II molecules. A set of 15 epitopes was initially selected based on predicted MHC binding capacity using the IEDB recommended 2.22 method. For each MHC considered, prediction percentile score (PR) thresholds of <10% (more restrictive) and <20% (less restrictive) were utilized to identify binders, where lower percentile scores correspond to higher predicted binding capacity. Selection also considered binding capacity as measured in *in vitro* binding assays based on the use of purified MHC (**Supplementary Table S1**, **Figure 1**). This analysis confirmed the 12 epitopes identified by previous observations on DRB1 (TEPITOPE and PROPRED) and DQ/DP (IEDB PR< 5%) and disclosed 3 additional epitopes. Epitopes 15-17-2018, 11-2018 and 17-2018 were predicted to be the most promiscuous (PR < 10%), (**Supplementary Table S1**, column 2) followed by the sequences 2-2014, 5-2018, 9-2018 and 15-2018 (PR < 20%) (**Supplementary Table S1**, column 3). **Supplementary Table S1** shows the number of HLA molecules bound by each epitope with PR < 10% (column 2) and PR < 20% values (column 3). These PR values were highly correlated (p < 0.0001, R = 0.880, R^2^ = 0.774). Epitope 11-2018 showed maximal affinity-binding to the DQA1* 05:01/DQB1* 02:01 allele (lowest PR % value = 0.08), while other 6 out of its 11 affinity values were ≤ 4.1% PR (**Supplementary Table S1**, **Figure 1**). In addition, most alleles interact with more than three epitopes. **Supplementary Table S1** lists the Percentil ranks values (PR) with which each epitope binds to each allele molecule. PR values lower to 10% (PR<10%), which correspond to a higher binding-affinity are shown in bold numbers, while PR<20% values, which correspond to a less restrictive and lower affinity-binding are shown with regular numbers. Therefore, **Supplementary Table S1** describes for instance, that the DQA1* 05:01/DQB1* 02:01 allele binds to 9 to epitopes with PR<10% and to one more, therefore, to 10 epitopes, with PR < 20%. The DQA1* 01:01/DQB1* 05:01 allele binds to 9 epitopes with PR<10% and to one more, therefore to 10 epitopes, with PR < 20%. The DRB1* 15:01 allele binds to 2 epitopes with PR<10% and to seven more, therefore, to 9 epitopes, with PR < 20%; and the DRB1* 13:02 allele binds to 5 epitopes with PR<10% and to three more, therefore, to 8 epitopes, with PR < 20%. Therefore, each one of these DQA1* 05:01/DQB1* 02:01, DQA1* 01:01/DQB1* 05:01, DRB1* 15:01 and DRB1* 13:02 molecules bind to 8-10 epitopes (**Supplementary Table S1**, **Figure 1**) suggesting that there is a depth of coverage of these alleles.

**Figure 2:**
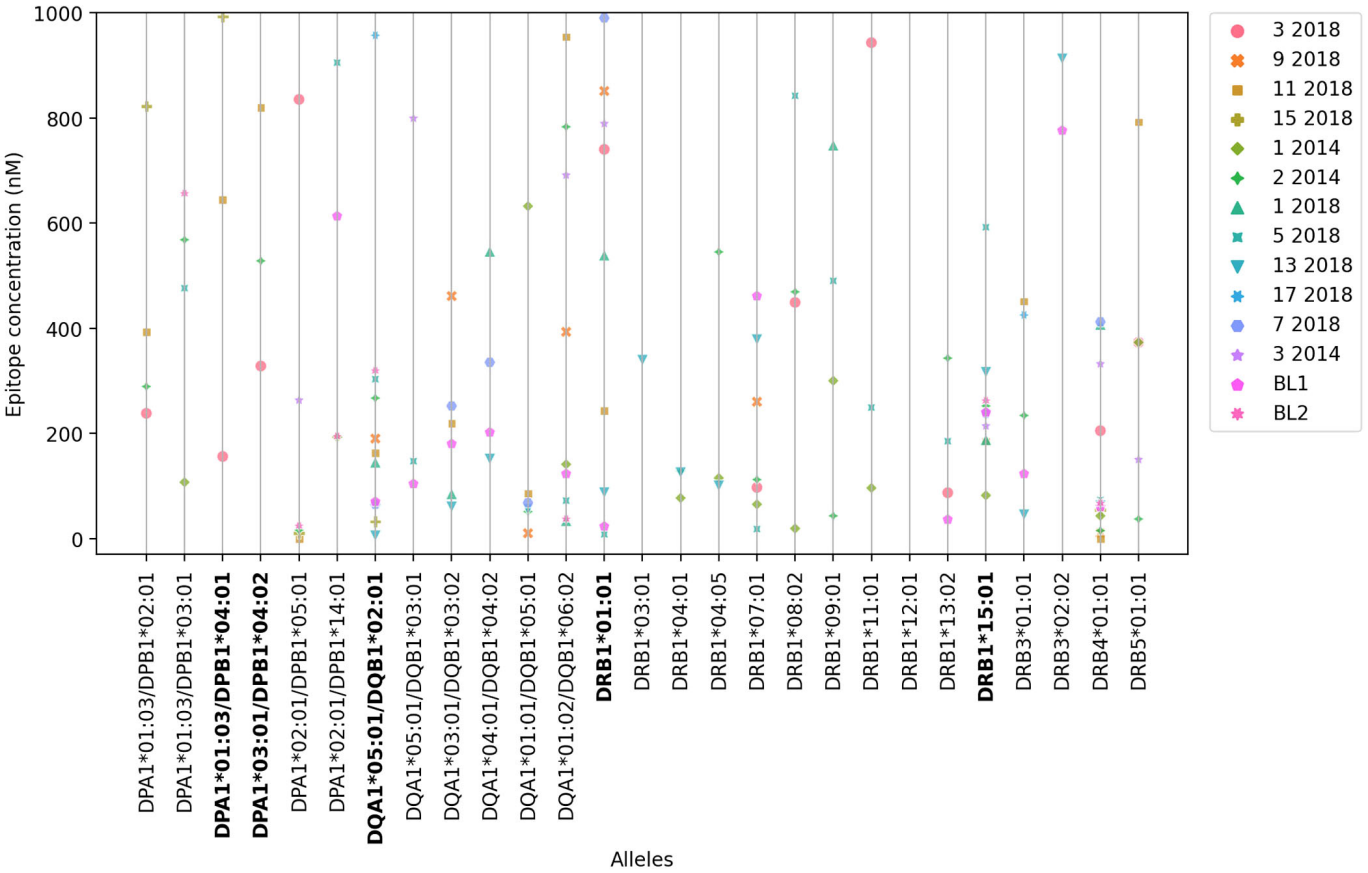
*In vitro* affinity-binding of synthetic NH36 epitopes for HLA DR, DQ and DP recombinant molecules The affinity of the epitopes to the HLA-DR,-DQ and-DP molecules *in vitro* is represented by their ability to inhibit the binding of a radioactive peptide probe to purified recombinant MHC molecules. The figure describes the IC50 ηM values developed when epitopes bind to 27 different HLA class II DPA1*/DPB1*, DQA1*/DQB1* and DRB1*, DRB3*, DRB4* and DRB5* most frequent molecules (IEDB HLA-class II tool). IC50 concentrations ≤ to 1000 ηM indicate the highest affinities of an epitope for each HLA receptor. The lower the IC50 concentration the stronger the epitope-HLA allele affinity.

Binding capacity was also assessed using *in vitro* assays based on the use of purified MHC molecules (**Supplementary Table S2**), based on their ability to inhibit the binding of a radioactive peptide probe to purified HLA molecules. IC50 concentrations ≤ to 1000 ηM, indicate the highest affinities of an epitope for each HLA receptor (**Supplementary Table S2**, values in bold). The lower the nanomolar value, the stronger the affinity of the epitope and HLA-molecule interaction. Most of the selected epitopes are promiscuous and bind to at least seven (25%) of the 27 HLA class II molecules tested (**Figure 2**, **Supplementary Table S2**). Epitopes 11-2018 and 5-2018 were both, predicted to bind 15 alleles (**Supplementary Table S1** column 3), and found to bind 13(48%-of alleles) in *in vitro* assays using purified MHC (**Supplementary Table S2**) and epitope 2-2014, predicted to bind 17 alleles (**Supplementary Table S1**, column 3) was determined to bind to 17 of the 27 tested HLA molecules (63%) (**Supplementary Table S2**, column 3, **Figure 2**). Epitope 11-2018 showed the highest promiscuity and affinity values (**Supplementary Tables S1, S2**; **Figures 1**, **2**).

HLA DPA1*01:03/DPB1*04:01 and DPA1*03:01/DPB1*04:02, which share similar binding specificity and are the most prevalent alleles in the vast majority of populations and geographic regions worldwide, each bound 3 of the epitopes in the panel with high affinity, and 4 unique epitopes between them. DQA1*05:01/DQB1*02:01, associated with high risk of VL (55, 56), was found to bind 11 of the epitopes with high affinity. DRB1*01:01 and DRB1*15:01, conversely predicted to be associated with resistance to VL, bound 9 and 10 epitopes, respectively (**Figure 2**, **Supplementary Table S2**); interestingly, a couple of cases of differential binding between DRB1*01:01 and DRB1*15:01 on the one hand, and DQA1*05:01/DQB1*02:01 on the other, could be noted, perhaps interesting targets for future studies.

### NH36 class II epitopes contain class I predicted ligands

Whether the selected class II epitopes also nest binders for HLA-A, HLA-B and HLA-C class I molecules was also assessed bioinformatically. This analysis is summarized in **Supplementary Table S3** showing the number of unique class I epitopes nested in each CD4 epitope (column 2); the number of unique class I alleles bound associated with them (column 3), the number of promiscuous peptides within each epitope (columns 4-11), and with which of the 37 alleles studied (16 alleles of HLA-A*, 11 alleles of HLA-B* and 10 alleles of HLA-C*) these epitopes may associate. The 11-2018 and 13-2018 epitopes predominate in these parameters, followed by 15-17-2018, BL2 and 7-2018.

### World HLA population coverage of NH36 epitopes

We next utilized the IEDBs population coverage tool to estimate the percent of individuals in the general worldwide population that could be expected to recognize the panel of epitopes. Coverage is determined considering that given epitope (or a nested subsequence) will elicit a response only in individuals that express an HLA molecule capable of binding that particular epitope. Accordingly, coverage is calculated on the basis of HLA genotypic frequencies and estimates the fraction of individuals expected to respond to a given epitope or sequence. For the present analysis, coverage at class I and class II *loci* were considered separately, and in both cases may represent underestimates of coverage as only alleles in the corresponding prediction reference panels were considered.

Eleven epitopes are estimated to be recognized by 50% or more of the general population on the basis class I alleles in the world population (**Supplementary Table S4**), and 14 of the 15 by class II. The 11-2018 and 13-2018 epitopes showed the highest coverage for class I (93.9% and 77.8% respectively), and class II molecules (92.7% and 85.9%, respectively), together with the 2-2014, BL1 and 5-2018 epitopes. The sequences of the epitopes 15-2018 and 17-2018 overlap by 16 amino acids. We combined them into a new epitope 15-17-2018 (**Supplementary Table S4**). This will not increase coverage, but it may increase coverage redundancy, which can be important if leakage occurs.

The coverage at class I *loci* considering the whole panel combined is >98%. The average number of epitope hits/HLA combination recognized by the population is approximately 11 (**Supplementary Figure S4**) and at least, in 90% of individuals, and the minimum number of epitope hits/HLA combinations recognized is 3.19 (PC_90_, **Supplementary Figure S4**). Class II panel coverage was similarly high (99.44%) though only based on DRB1, DQB1 and DPB1, as there is no reliable data on the frequency of DRB3/4/5 alleles (though they are in strong linkage with various DRB1 alleles (**Supplementary Table S4**, **Supplementary Figure S5**). For class II we note an average number of epitope hits/HLA combination recognized by the population of 17.15 and at least 7.67 of epitopes/allele combinations recognized by 90% of the world population (PC_90_) (**Supplementary Figure S5**). Thus, while coverage for NH36 epitopes is slightly higher for class II than for class I (**Supplementary Figures S4, S5**), it is very good in both cases.

### NH36-epitopes bind to HLA molecules correlated with VL susceptibility or resistance

The common HLA class II alleles DRB1*15:01 and DRB1*01:01, as well as the less common alleles DRB1*15:02 and DRB1*16:02, have been associated with a VL resistant/protective phenotype (55, 56) (see **Supplementary Tables S5, S6**). As shown in **Supplementary Table S2**, 12 epitopes in our panel bound either DRB1*15:01 or DRB1*01:01 with high affinity (IC50 <1000 ηM) in *in vitro* binding assays; 7 of these peptides (11-2018, 1-2018, 5-2018, 13-2018, 7-2018, 3-2014 and BL1) bound both alleles.

Another allele, HLA-DQB1*05:01, is associated with intermediate VL risk (56) (**Supplementary Table S6**). Six of the epitopes bound to DQB1*05:01 with high affinity (**Supplementary Table S2**). Interestingly, all these peptides also bound DRB1*15:01 and/or DRB1*01:01. Finally, eleven epitopes were determined to bind DQB1*02:01, associated with high risk of VL (55, 56) (**Supplementary Table S6**, **Figure 2**), with high affinity (**Supplementary Table S2**).

Taken together, this analysis reinforces that the NH36 epitopes could protect not only individuals with natural resistance or protection phenotypes, but also those susceptible or at risk of contracting VL.

### NH36 epitopes are recognized by IgG, IgG1, IgG2, IgG3 and IgG4 antibodies

**Figure 3:**
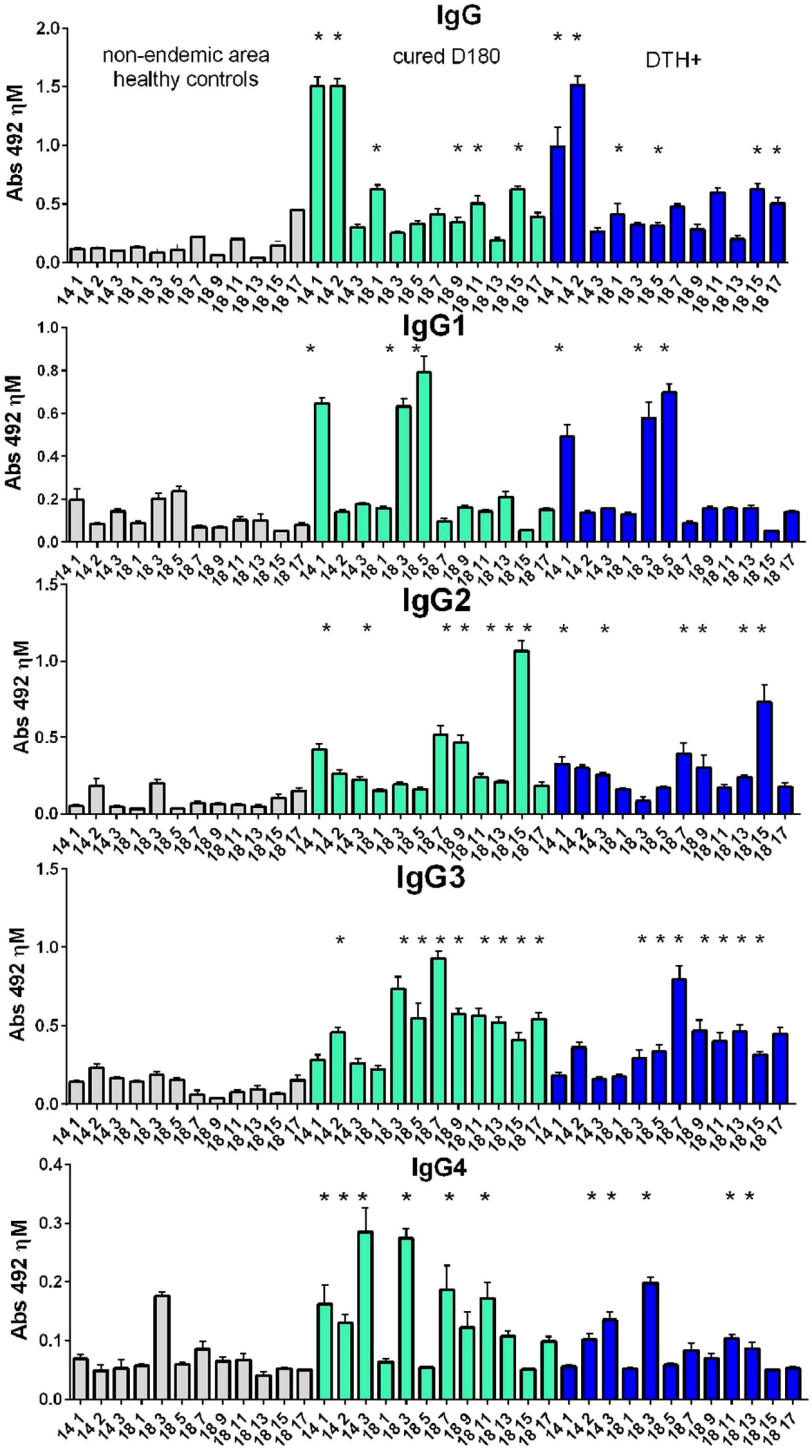
IgG, IgG1, IgG2, IgG3 and IgG4 antibody responses to NH36 epitopes. Analysis of serum humoral response to NH36 epitopes. Anti-IgG, IgG1, IgG2, IgG3 and IgG4 antibody levels against epitopes 1-2014, 2-2014, 3-2014, 1-2018, 3-2018, 5-2018, 7-2018, 9-2018, 11-2018, 13-2018, 15-2018 and 17-2018 were evaluated through an ELISA assay using 1/100 diluted sera from individuals cured of VL (n=10), asymptomatic DTH+ contacts (n=10) and healthy controls from a non-endemic area (n=7). Asterisks represent significant differences between the absorbance values of patients and their respective healthy controls (ANOVA) obtained using the Sidak test for multiple comparisons.

The results indicate that the *Leishmania (L.) infantum chagasi* infection induced a humoral response against the NH36 peptides. Anti-NH36 epitope antibody responses in sera of controls were lower and significantly different from those of cured and DTH^+^ individuals (**Figure 3**). The highest IgG response was directed against epitopes 1-2014 and 2-2014, and the highest IgG1 response against epitopes 1-2014, 3-2018 and 5-2018. Furthermore, the predominant IgG2 response was directed against epitope 15-2018, followed by 7-2018, 9-2018 and 1-2014. In the IgG3 response, epitopes 3-2018 and 7-2018 also stood out, followed by the other epitopes from 2018, as well as 2-2014. Finally, the strongest IgG4 response was detected towards epitopes 3-2014 and 3-2018, followed by 7-2018 and 9-2018.

### NH36 epitopes induce intracellular production of cytokines in CD4^+^ and CD8 T lymphocytes

**Supplementary Figure S1** summarizes the ICS gating strategy. The percentage of CD3^+^ T lymphocytes stimulated with NH36 epitopes is similar in the healthy control groups, patients cured of VL (D180) and asymptomatic (DTH^+^), with mean frequencies of 70%, and a slight increase in patients cured (D180) (**Supplementary Figure S6**).

**Figure 4:**
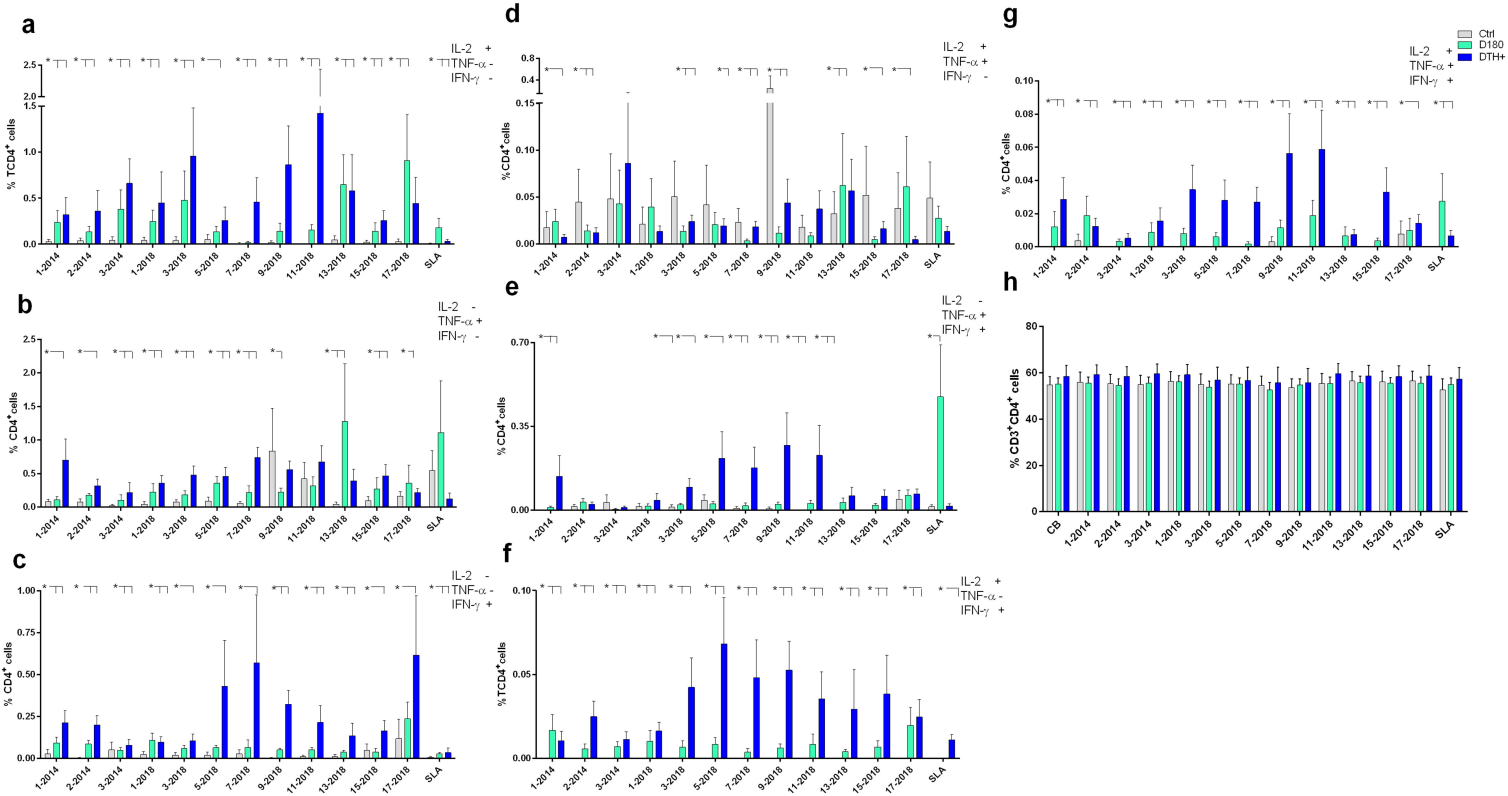
Intracellular production of cytokines in response to NH36 epitopes by CD4^+^ lymphocytes. PBMC of individuals cured of VL (n=10), asymptomatic DTH+ contacts (n=10) and healthy controls from a non-endemic area (n=7) were incubated with the NH36 epitopes (25 µg/ml) or with SLA (10 µg/ml). The ICS method with the Boolean analysis allowed the identification of CD4^+^ T cells producing one cytokine [IL-2 **(a)**, TNF-α **(b)** or IFN-γ **(c)**], two cytokines [IL-2 and TNF-α **(d)**, TNF-α and IFN-γ **(e)** and IL-2 and IFN-γ **(f)**] or three cytokines simultaneously (IL-2, TNF-α, IFN-γ **(g)**, the multifunctional cells, which correlate with the induction of protection (62). The frequencies of CD3^+^CD4^+^ lymphocytes were also assessed **(h)**. Results are expressed as means + SE and analyzed with the 95% confidence interval test. Asterisks and lines compare means with the healthy controls.

**Figure 5:**
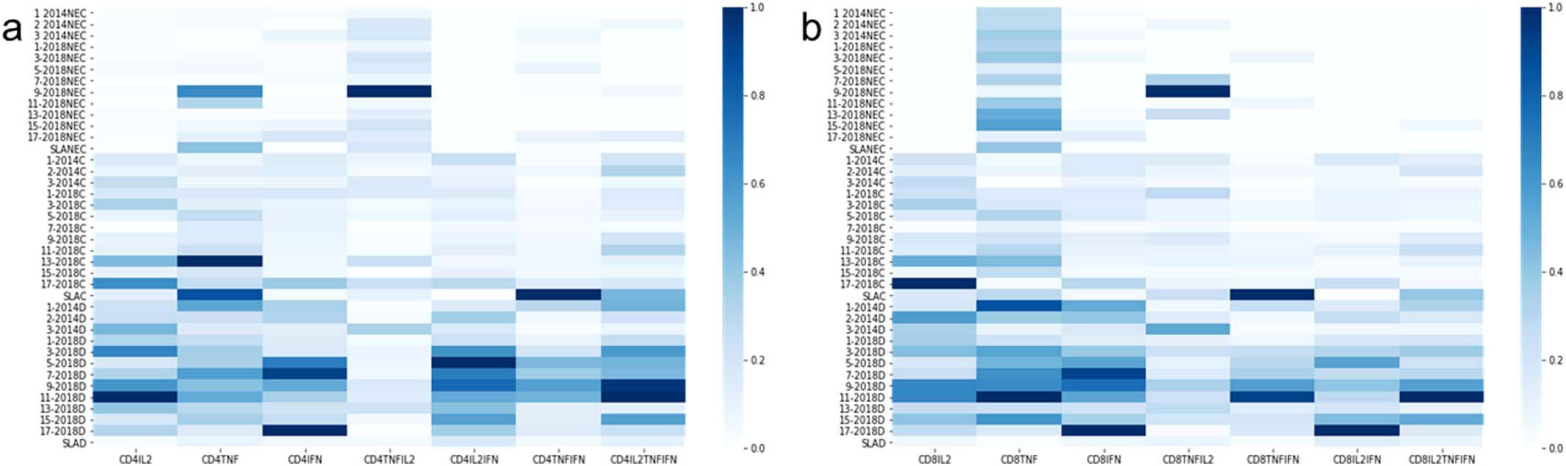
Heatmaps of the mean frequency values of cytokine-producing CD4^+^ and CD8^+^ T lymphocytes. Heatmaps were generated based on the mean values of the frequencies of cytokine-producing CD4^+^ **(a)** and CD8^+^ **(b)** T lymphocytes to help to identify the most potent epitopes. In this case, we used a graph normalized by columns to facilitate the visualization of the most potent epitopes, even for the case of cell subtypes that appear less frequently, such as triple-secretory and multifunctional.

The frequencies of CD4^+^ T lymphocytes producing only IL-2, TNF-α or IFN-γ (**Figures 4a–c**, respectively) were predominantly higher in DTH^+^ than in cured individuals, and higher in these than in controls, except for the 9-2018 and the 13-2018 epitopes, which increased the proportions of CD4^+^TNF-α^+^ lymphocytes in controls and in DTH^+^ subjects, respectively (**Figure 4b**). All epitopes are more potent than the soluble SLA antigen in increasing proportions of IL-2 or IFN-γ producing CD4^+^ T lymphocytes (**Figures 4a, c**). Epitopes 3, 9 and 11-2018 stood out in the increase of the frequencies of CD4^+^IL-2^+^ T lymphocytes (**Figure 4a**); epitopes 1-2014, 7 and 13-2018 of the frequencies of CD4^+^TNF-α^+^ T cells (**Figure 4b**), and epitopes 5, 7, 9 and 17-2018 of the proportions of CD4^+^IFN-γ^+^ T cells (**Figure 4c**). Additionally, healthy controls showed higher frequencies of IL-2 and TNF-α double-producers and TNF-α single-producers than cured or DTH subjects (**Figure 4d**) mainly in response to epitope 9-2018. In contrast, CD4^+^ lymphocytes producing TNF-α and IFN-γ had their frequencies increased in DTH^+^ individuals, mainly in response to epitopes 1-2014 and 5, 7, 9 and 11-2018 (**Figure 4e**). Only the SLA antigen raised these proportions in cured patients. The frequencies of IL-2 and IFN-γ producing CD4^+^ T lymphocytes were predominant in DTH^+^ individuals, mainly in response to epitopes 2-2014 and 3 to 17-2018 (**Figure 4f**). Finally, the frequencies of triple-secreting or multifunctional TCD4^+^ T lymphocytes were mainly increased in DTH^+^ individuals, especially against epitopes 9 and 11-2018 followed by sequences 1-2014, and 3, 5, 7 and 15 from 2018. The response to the SLA antigen was higher in cured individuals (**Figure 4g**), as it was detected in lymphocytes secreting TNF-α^+^ and IFN-γ^+^ (**Figure 4e**). The frequencies of CD3^+^CD4^+^ lymphocytes were similar in all groups of individuals (**Figure 4h**). Additionally, a heatmap was generated based on the mean values of the frequencies of cytokine-secreting CD4^+^ T lymphocytes (**Figure 5a**) to help identify the most potent epitopes. Epitopes 9, 11, predominantly increased the frequencies of multifunctional cytokine triple-producers cells of cured patients (C) and more, of DTH^+^ subjects (D) (**Figure 5a**).

**Figure 6:**
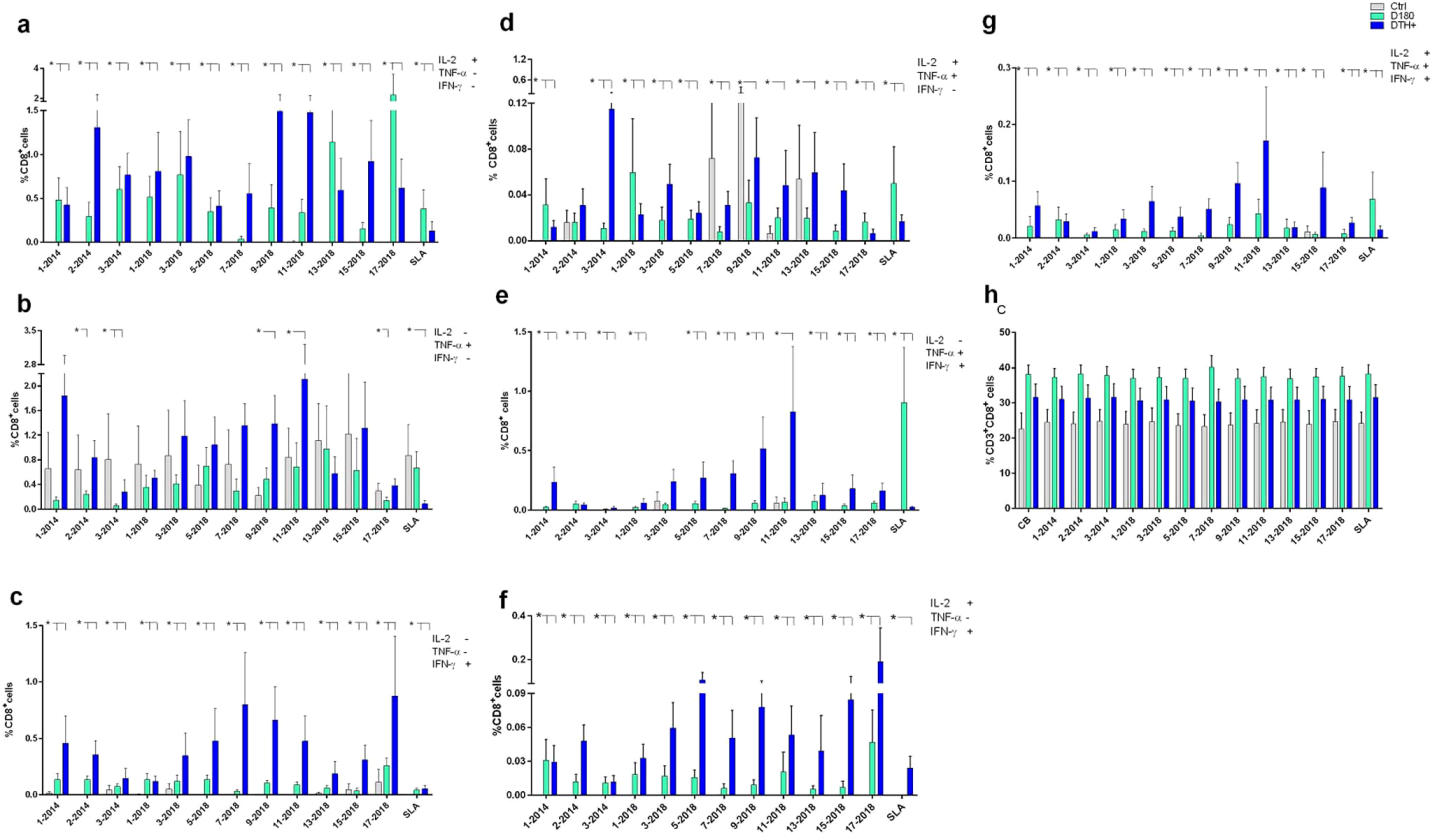
Intracellular production of cytokines by CD8^+^ lymphocytes in response to NH36 epitopes. PBMC of individuals cured of VL (n=10), asymptomatic DTH+ contacts (n=10) and healthy controls from a non-endemic area (n=7) were incubated with the NH36 epitopes (25 µg/ml) or with SLA (10 µg/ml). The ICS method with the Boolean gate analysis allowed the identification of CD8^+^ T cells producing one cytokine [IL-2 **(a)**, TNF-α **(b)** or IFN-γ **(c)**], two cytokines (IL-2 and TNF-α **(d)**, TNF-α and IFN-γ **(e)** and IL-2 and IFN-γ **(f)** or three cytokines simultaneously (IL-2, TNF-α, IFN-γ **(g)**, the multifunctional cells, which correlate with the induction of protection (62). The frequencies of CD3^+^CD8^+^ lymphocytes were also assessed **(h)**. Results are expressed as means + SE and analyzed with the 95% confidence interval test. Asterisks and lines compare means to the healthy controls.

The response of CD3^+^CD8^+^ T lymphocytes to the NH36 epitopes was studied as well. The frequencies of single-producers of IL-2 or IFN-γ were, as detected for CD4^+^ T cells, predominantly higher in DTH^+^ individuals than in cured and controls (**Figures 6a, c**). In the case of TNF-α production, epitopes 9 and 11 stood out, with increases of 84% and 60%, respectively, in these proportions in DTH^+^ individuals (**Figure 6b**). However, controls also responded with low frequencies against most epitopes. Furthermore, the joint secretion of IL-2 and TNF-α was greater in cured patients and DTH^+^, with the exception of epitopes 7, 9 and 13-2018 (**Figure 6d**). The most potent epitope was 3-2014.

In contrast, CD8^+^ T lymphocytes frequencies were increased in DTH^+^ individuals, in TNF-α and IFN-γ producing cells, mainly in response to epitopes 9 and 11-2018 (**Figure 6e**), and in IL-2 and IFN-γ producing cells, to epitopes 5, 9, 15 and 17-2018 (**Figure 6f**). Finally, as described for CD4^+^ T lymphocytes (**Figure 4g**), it was possible to detect increases in frequencies of triple-producers of cytokines or multifunctional CD8^+^ T lymphocytes, in cured individuals which were stronger, especially in DTH^+^ individuals, promoted by most epitopes, especially epitopes 9 and 11-2018 (**Figures 5b**, **6g**). The response to the SLA antigen was, as described for multifunctional CD4^+^ T lymphocytes (**Figure 4g**), greater in cured individuals, for the multifunctional (**Figure 6g**), IL-2 single-producers (**Figure 6a**) and IL-2 and TNF-α^+^ (**Figure 6d**) and TNF-α^+^ and IFN-γ^+^ double-producers (**Figure 6e**). The percents of CD3^+^CD8^+^ T cells (**Figure 6h**) were slightly higher for DTH^+^ subjects.

### NH36 epitopes promote cytokine secretion by PBMC

**Figure 7:**
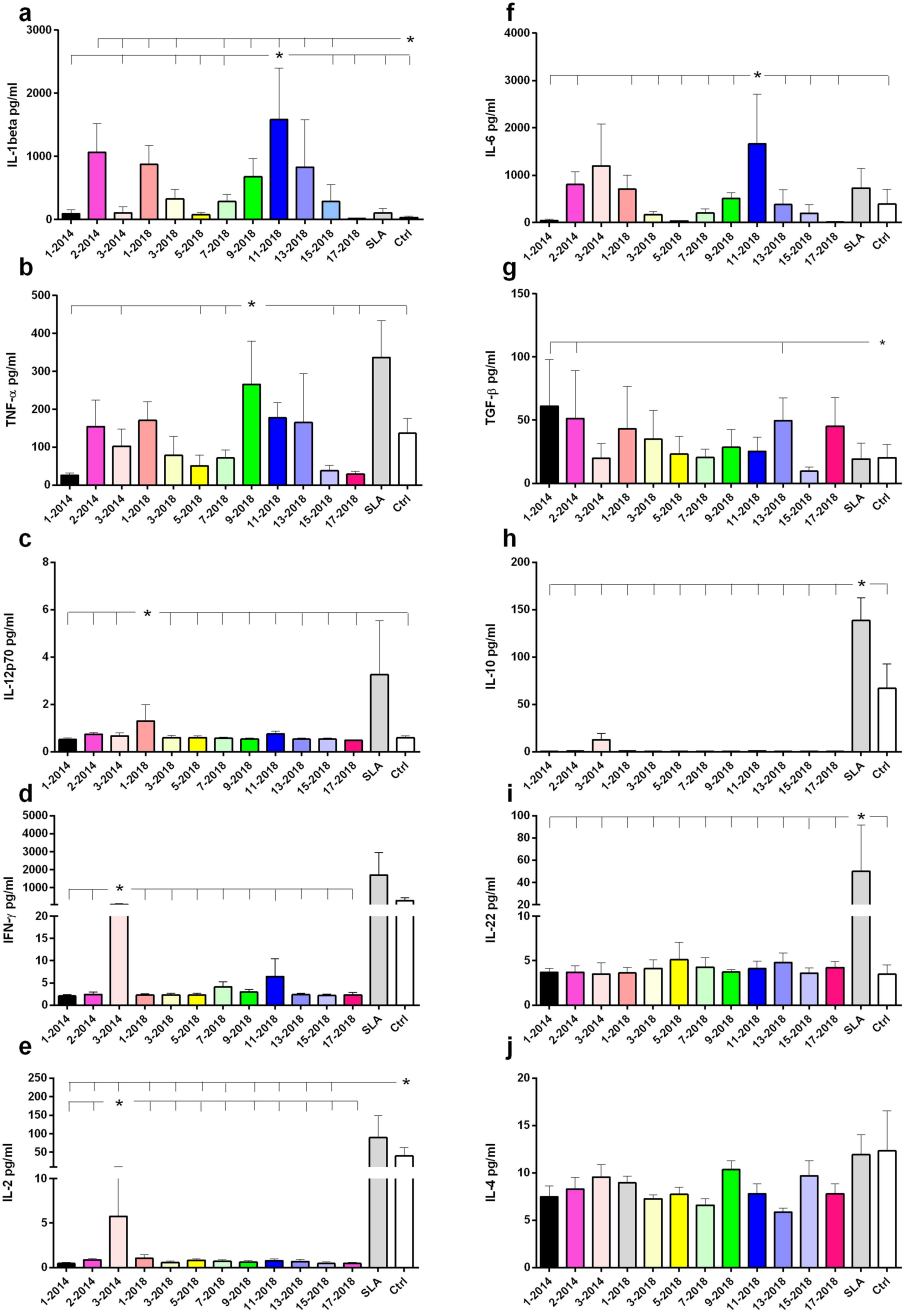
Cytokine secretion in response to the NH36 epitopes. Cytokine levels produced by PBMC from DTH+ individuals stimulated *in vitro* with NH36 epitopes were assessed. PBMCs from asymptomatic individuals (n=6) were incubated with 25 µg/ml of NH36 epitopes: 1-2014, 2-2014, 3-2014, 1-2018, 3-2018, 5-2018, 7-2018, 9- 2018, 11-2018, 13-2018, 15-2018 and 17-2018, with soluble *Leishmania* antigen (SLA) or without stimulus (base control) for a period of 72 hours and the IL-1β **(a)**, TNF-α **(b)**, IL-12p70 **(c)**, IFN-γ **(d)**, IL-2 **(e)**, IL-6 **(f)**, TGF-β **(g)**, IL-10 **(h)**, IL-22 **(i)**, IL-4 **(j)** cytokine secretion was measured in the supernatant using the Luminex assay. For statistical analysis, the 95% CI test was used. Asterisks and lines compare means of the most potent epitopes to the other epitopes and controls.

The secretion of cytokines in supernatants of PBMC previously stimulated with NH36 epitopes was evaluated in the DTH^+^ group, which showed the best multifunctional T lymphocyte responses. IL-1β secretion was significantly increased in response to the 11-2018, 9-2018, 13-2018, 2-2104 and 1-2018 epitopes, in comparison to the basal control and SLA (**Figure 7a**). An increase in TNF-α secretion was also observed in response to the 9-2018 epitope when compared to the basal control (**Figure 7b**). As for the production of IL-12p70, it was slightly increased in response to epitope 1-2018 if compared with the other epitopes and with the basal control (**Figure 7c**). Finally, only epitope 3-2014 promoted increases in IFN-γ and IL-2 secretion, but at levels similar to the control (**Figures 7d, e**, respectively).

Secretion of the anti-inflammatory cytokine IL-6 was higher in response to the 11-2018 epitope (**Figure 7f**). Additionally, 1-2014, 2-2014 and 13-2018 produced more TGF-β than SLA and the basal control (**Figure 7g**). In contrast, only SLA and control (**Figure 7h**), or SLA alone (**Figure 7i**) stimulated the secretion of IL-10 and IL-22, respectively. No secretion of IL-4 was observed (**Figure 7j**). In conclusion, regarding the pro-inflammatory response, epitope 9 stood out in TNF-α secretion, and epitope 11 along with 9, 7, 1, 3, 13 and 15 of 2018 and 2, 3 of 2014, in IL-1β secretion. Also suggesting a regulatory response, epitope 11-2018 promoted, in addition to IL-1β, secretion of IL-6.

### Correlation between HLA DNA typing and susceptibility to VL

HLA-DNA typing for 11 *loci* (HLA-A*, HLA-B*, HLA-C*, DRB1*, DBQ1*, DRB3*, DRB4*, DRB5*, DQA1*, DPA1*, DPB1*) was determined in 8 cured VL patients and 10 DTH^+^ subjects (**Supplementary Table S7**). The HLA-C*04:01 allele was found in 6 of 8 cured individuals, but in none of the 10 DTH^+^ individuals (p = 0.0043), suggesting its correlation with susceptibility to human VL. Although the group of patients studied was relatively small, it was possible to observe that the alleles associated with protection, DRB1*0101 and DRB1*1501 (55, 56), considered together, were present in 4 of the 10 DTH^+^ patients, but were absent in the 8 cured patients (*x* ^2^ = 0.0023) (**Supplementary Table S7**). Noteworthy, 3 DTH^+^ subjects have in their phenotype the allele DRB1*01:01, related to protection against VL (CY:5, CY:7 and CY:9) but also DQB1*05:01, associated with intermediate risk (**Supplementary Tables S7, S8**) (56); a fourth subject DTH^+^ (CY:1) showed the DRB1*1501 allele. In contrast, the DRB1*13:02, DRB1*13:01 and DRB1*02:01, all associated with high risk, were only detected in 3 VL patients (BY:1, BY:9 and BY:10) (**Supplementary Tables S7, S8**). In addition, the high risk alleles DRB1*10:01 and DRB1*11:01 were present in 3 DTH^+^ subjects (CY:4, CY:5 and CY:8) and 2 VL patients (BY:6 and BY:7) (**Supplementary Tables S7, S8**) (55). **Supplementary Table S8** also summarizes the individual response of the patients to the epitopes. Most of the epitopes (1–11) promoted the desired increase of the frequencies of multifunctional IL-2^+^TNF-α^+^IFN-γ^+^ secreting-CD4^+^ and CD8^+^ T cells (**Supplementary Table S8**), confirming that they can be useful in the generation of a memory protective immune response both in individuals naturally resistant and highly susceptible to VL.

### Analysis of epitope conservancy

The percentage identity of the epitopes in *L. (L.) donovani* was found to be quite high overall (range 98.75-100%; median = 99.41%), though slightly lower than 100%, indicating a mild variability in the NHs sequences of the 16 genomes (**Supplementary Table S9**). *Leishmania (L.) infantum* and *Leishmania (L.) infantum chagasi*, also of the VL complex, showed 99.6% conservation and *L. (L.) tropica* and *L. (L.) major*, agents of Old World CL showed 99.7% and 98.2% identity, respectively. Indicating phylogenetic distance, identity was lower for *L. (L.) tarentolae* (93.1%), a lizard parasite, and for *L. (V.) panamensis*, *L. (V.) braziliensis* and *L. (V.) guyanensis*, agents of New World CL of the *Viannia* subgenus (83.97% to 85.3%), despite their fewer genomes analyzed (**Supplementary Table S9**). The high level of cross-identity supports the use of NH36 multiepitope vaccines in generation of universal protection against both VL and CL.

### Design of multiepitope constructs

**Figure 8:**
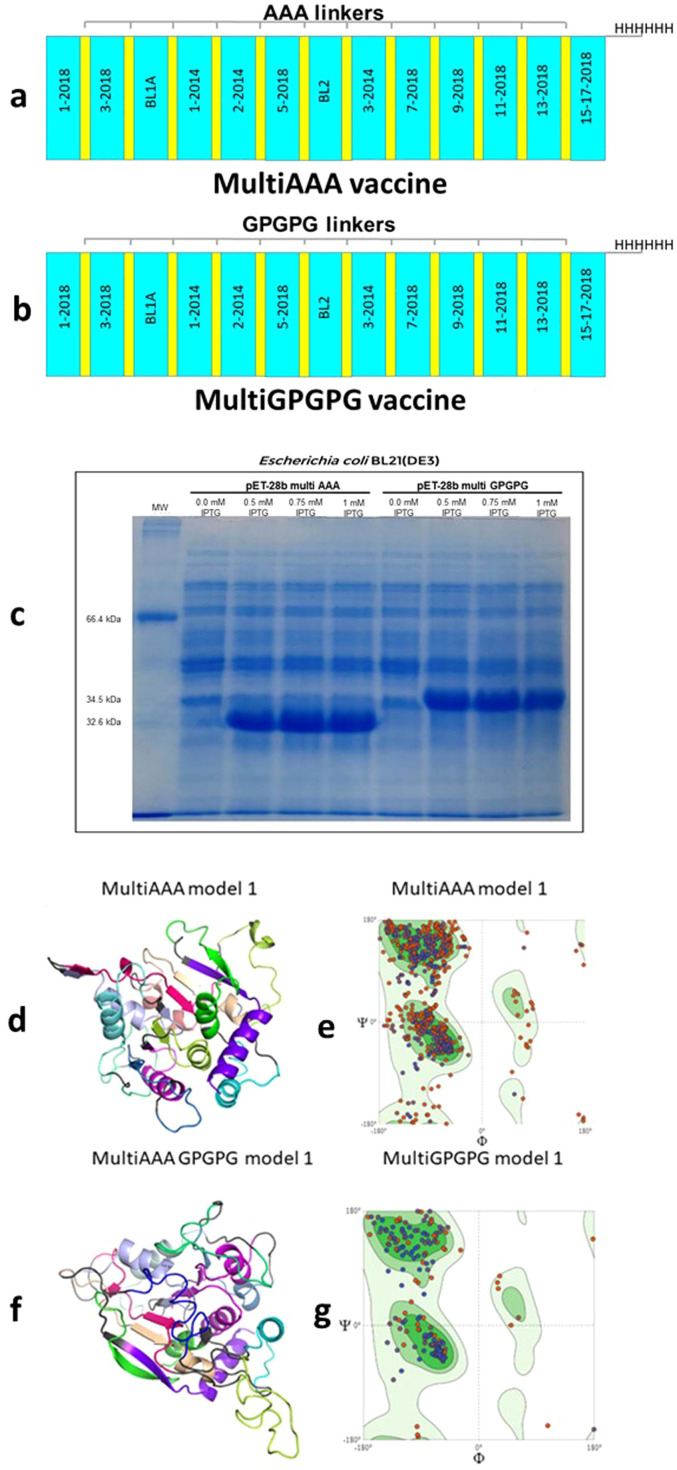
MultiAAA and MultiGPGPG proteins schematic representation, expression, tertiary structure models and validation. Schematic representations of the MultiAAA **(a)** and MultiGPGPG **(b)** multiepitope proteins. Each protein is composed of the 13 NH36 epitopes (1-2014, 2-2014, 3-2014, 1-2018, 3-2018, 5-2018, 7-2018, 9-2018, 11-2018, 13-2018, 15-17-2018, BL-1 and BL-2). Twelve out of the thirteen epitopes were genetically modified by the addition of the AAA spacer in the MultiAAA, or GPGPG spacer, in the MultiGPGPG protein, and the last epitope was linked to a tail of 6 Histidines. Each protein represents a combination of “helper” CD4^+^ T cell epitopes, which also contains sequences of epitopes for CD8^+^ T cells. MultiAAA and MultiGPGPG were cloned in pET28b and expressed by *E coli* BL21(DE3) using 0.5 mM, 0.75 or 1 mM IPTG or no induction. The bacterial lysate was resuspended in Laemmli sample buffer and submitted to 12% SDS-PAGE under denaturing conditions **(c)**. Molecular modelling of the tertiary structures of MultiAAA **(d)** and Multi GPGPG **(f)** proteins were obtained out using the Swiss-Model tool, by homology with the pdb of the Cristal Structure of the Nucleoside Hydrolase of *Leishmania major* (1EZR) (RCSB PDB tool) and the Pymol 2.5.7 program for Graphic presentation. Refinement of the models by the Swiss Model and the PROCHEK tool of PDBSUM indicated the Model 1 of each proteins **(d, f)**. For model validation, the Ramachandran plots of MultiAAA Model 1 **(e)** and MultiGPGPG Model 1 **(g)** were obtained.

Two multiepitope vaccine constructs were designed. In order to avoid the generation of neoepitopes, epitopes in the MultiAAA construct were linked using three Alanines (AAA) as spacers; in the MultiGPGPG construct epitopes were linked with Glycine-Proline-Glycine-Proline-Glycine (GPGPG) spacers. Epitope positions in the vaccines followed their natural sequence order in the NH36 protein (**Figures 8a, b**). The 15-17-2018 final epitope is followed by a 6 Histidine tag, which facilitates the purification of the expressed peptide.

The MultiAAA vaccine (mw = 32205.23 D) (**Figure 8a**) has 310 amino acids (29 negatively and 23 positively charged) and a theoretical pI of 6.15. It showed an estimated half-life of 20 h in mammalian reticulocytes *in vitro*, 30 minutes in yeast *in vivo* and over 10 hours in *Escherichia coli in vivo*. Its instability index is 24.38, which classifies it as a stable protein. Its aliphatic index is 107.13 and mean hydropathic index (GRAVY) is 0.422. The MultiGPGPG vaccine (mw = 34445.43 D) (**Figure 8b**) is composed of 339 amino acids (29 negatively and 23 positively charged), has a theoretical pI of 6.15, and an estimated half-life in mammalian reticulocytes *in vitro* of 20h, in yeast *in vivo* of 30 min and in *Escherichia coli in vivo*, greater than 10h. Its instability index is 21.59, which classifies it as a stable protein. Its aliphatic index is 86.49 and its GRAVY is 0.010. MultiAAA and MultiGPGPG were predicted as non-toxic by (ToxinPred2 ML and Hybrid scores = 0.23 and 0.19, respectively and Merci and Blast = 0) and BTXPred tools and as non-allergens.

### Secondary and tertiary structure models, validation, refine and cloning

Both proteins were cloned in the pET-28b (+) vector (**Supplementary Figure S7**) and expressed by *E. coli* BL21 (DE3) (**Figure 8c**). The MultiAAA protein has an estimated 45% of α-helix, 13% of β-strand and 42% of coils. MultiGPGPG protein holds an estimated of 31% of α-helix, 9% of β-strand and 60% of coil domains (**Supplementary Figure S8** **).** The Swiss Model tool (SM) provided three 3D models for the MultiAAA vaccine. SM and PROCHEK-PDBSUM indicated Model 1 (**Figure 8d**) for its best Q mean Z score, highest Qmean Disco Global, second higher GMQE and good agreement with the usual protein models (overall G factor) (**Supplementary Table S10**). The MultiAAA Model 1 Ramachandran graph disclosed 86.96% of the residues in a favored position, 4.18% outliers and 3.92% rotamers (**Figure 8e**). SM disclosed also two 3D models for MultiGPGPG. Model 1 showed the best Q mean Z score, highest Qmean Disco Global and highest GMQE (**Figure 8f**) and its Ramachandran graph showed 87.23% of the residues in a favored position, 3.04% outliers and 6.53% rotamers (**Figure 8g**). Despite the lack of signal peptides in their N-terminal ends, potential glycosylation sites were disclosed at positions 31 (NQTL, 0.6854), 82 (NASQ, 0.6530) and 214 (NVTM, 0.6854) of the MultiAAA, and positions 33 (NQTL, 0.6756), 88 (NASQ, 0.6266) and 238 (NVTM 0.6796) of the MultiGPGPG protein.

### Prediction of the immune stimulation responses to the multiepitope vaccines

Potential immunogenicity of both constructs was evaluated bioinformatically using the C-ImmSim tool, as detailed in the methods. Both constructs displayed similar predicted results. Specifically, IgM and IgM+IgG with major IgG1 and lower IgG2 antibody titers were predicted, progressively increasing from primary to secondary humoral responses (**Supplementary Figure S9A, S10A**). The B cell populations are predicted to be high, sustained, and active along the year (**Supplementary Figures S9B, C, S10B, C**). Remarkably, Th1 T cell values are predicted to increase after vaccination and memory Th cells to be persistent during the whole year (**Supplementary Figures S9D–F, S10D–F**). Macrophage activity should be high (**Supplementary Figures S9H, S10H**). IL-2 was predicted as the most secreted cytokine, with mildly higher counts for MultiAAA (**Supplementary Figures S9I, S10I**), followed by IFN-γ and IL-12 confirming the raise a Th1 pro-inflammatory response. Lower predicted secretion of TGF-β and IL-10 suggests a mild regulatory response while secretion of IL-6 was not observed (**Supplementary Figure S9I, S10I**). In contrast, active cytotoxic T cells may decay, more rapidly for MultiGPGPG (**Supplementary Figure S10G**) than for MultiAAA (**Supplementary Figure S9G**). The negative control should not promote any antibody response and show only B IgM activated T cells (**Supplementary Figures S11A, B**). No Th1, Th2, T memory, Th17 and resting macrophage responses and only a very low Treg and resting T cells (**Supplementary Figures S11D–F, H**) are predicted. Peaks of IFN-γ alone were observed after injections. In contrast, similar results to those of the multiepitope vaccines were obtained for the positive control (A4H9H8.1 protein) (**Supplementary Figures S12A-I**) differing only by a higher IL-2 secretion than MultiGPGPG.

### MultiAAA and MultiGPGPG proteins promote the increase of expression of activation induced markers and intracellular production of cytokines by CD4^+^ and CD8^+^ T cells

**Figure 9:**
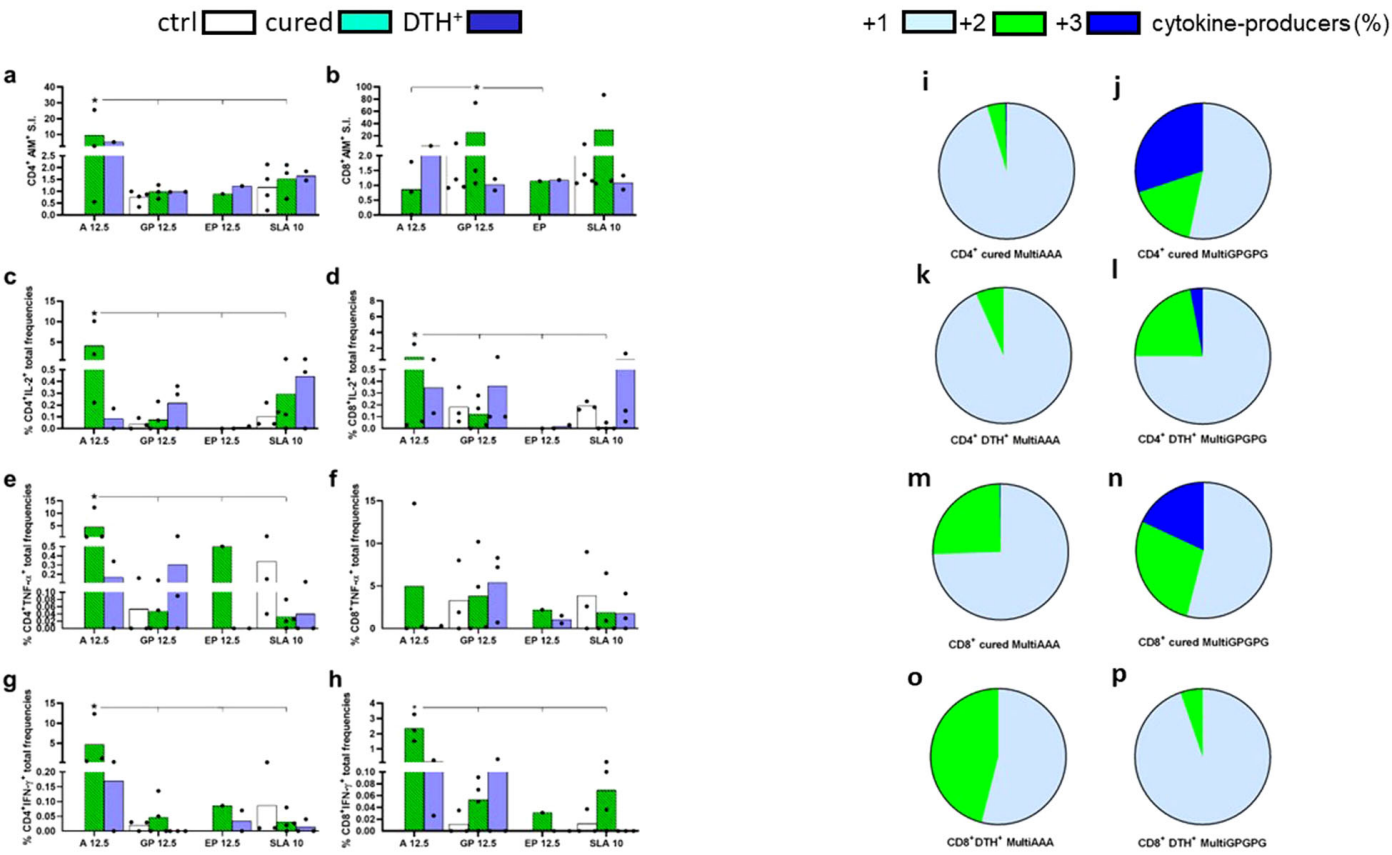
*In vitro* CD4^+^ and CD8^+^ T cell responses to the MultiAAA and MultiGPGPG recombinant purified proteins. PBMCs of cured VL patients (n = 4) (cured), asymptomatic DTH+ individuals (n = 2-3) (DTH^+^) and healthy blood donors of the endemic area of Sergipe (n = 4) (ctrl) were incubated with 12.5 µg/ml of MultiAAA or MultiGPGPG proteins, 12.5 µg/ml of the synthetic epitope mixture (0.96 ug/ml of each epitope), 10 µg/ml SLA or with no addition, as described in Methods. Increased expression of activation induced markers (AIM) in CD4^+^CD69^+^ CD40L^+^ **(a)** and CD8^+^CD69^+^CD137^+^ T cells **(b)** were calculated as fold-change values for each individual in response to the antigens, over its own background values, with no antigen addition. Total frequencies of IL-2, TNF-α and IFN-γ-producing CD4^+^ (**c, e, g**, respectively) and CD8^+^ T cells (**d, f**, **h,** respectively), in response to MultiAAA and MultiGPGPG proteins, were recorded by the ICS protocol described in Methods. For ICS experiments, the frequencies of antigen-stimulated lymphocytes of each patient were discounted from the background values of its PBMC incubated with no antigen addition. Means of AIM and ICS groups of treatments are represented by columns, and individual results by dots. Asterisks and lines compare the responses to the different antigens by the 95% confidence interval test (IC95%). The percentage contribution of CD4^+^ **(i–l)** and CD8^+^ T cells producers **(m–p)** of one cytokine (IL-2, TNF-α and IFN-γ), any combination of two or three cytokines, in response to MultiAAA or MultiGPGPG antigens, was calculated from the ICS results obtained from cured and DTH^+^ subjects by the IC95% test.

PBMC of cured and asymptomatic subjects were incubated with MultiAAA and MultiGPGPG purified recombinant proteins. Purity of the MultiAAA and MultiGPGPG preparations was confirmed by SDSPAGE analysis (**Supplementary Figure S3**). Despite the small number of individuals, as a proof of concept, the overall AIM and ICS results indicate that the two multiepitope vaccines were capable of triggering T cell immune responses (**Figures 9a–h**). In fact, the MultiAAA protein promoted the highest CD4^+^ responses by AIM (9.6 fold-increase) (**Figure 9A**), and the highest total frequencies of IL-2 (**Figure 9c**), TNF-α (**Figure 9e**) and IFN-γ-producing CD4^+^ T cells (**Figure 9g**). In contrast, the MultiGPGPG protein predominated in the CD8 response by AIM (25 fold-increase) (**Figure 9b**) and was not different from the MultiAAA in total frequencies of CD8^+^TNF-α lymphocytes (**Figure 9f**), while the Multi AAA was stronger in total frequencies of CD8^+^IL-2^+^ and CD8^+^IFN-γ^+^-producing T cells (**Figures 9d, h**, respectively). Responses of the heathy blood donor controls of endemic area were negative or lower than those promoted by MultiAAA and MultiGPGPG, with the exception of total frequencies of CD4 and CD8 T cells producing TNF-α (**Figures 9e, f**) and, total frequencies of CD8 T cells producing IFN-γ (**Figure 9g**), in response to SLA antigen, suggesting that even these blood donors, who tested negative for the presence of the cross-reactive Chagas Disease antigen can react with the mixture of total *Leishmania* proteins that compose the SLA antigen.

Additionally, the functional profile of cytokine-producing T cells was calculated as the percentage of cells producing one, any combination of two or three cytokines by ICS (**Figures 9i–p**). As detected by IC95%, both in the cured and DTH^+^ individuals, the MultiGPGPG protein promoted the most increased differentiation of the single-producers into double- and triple-producers multifunctional CD4^+^ T cells, which secret IL-2, TNF-α and IFN-γ, suggesting that MultiGPGPG induces stronger Th1 responses than MultiAAA (**Figures 9i–l**). Likewise, MultiGPGPG enhanced the frequencies of double-producers and multifunctional CD8^+^ T cells of cured individuals (**Figures 9m, n**) but was less potent than MultiAAA (**Figures 9o, p**) in double-cytokine producers of DTH^+^ subjects.

## Discussion

While VL constitutes a severe public health threat, there is no currently licensed human vaccine against it (18). Several factors could be responsible for that delay (18). Considering the lethality, severity, high toxicity and rate of chemotherapy failure, and long period of hospitalization, the development of a preventive vaccine is highly needed and supported (17, 18, 71). However, while intensive vaccine research was performed in the murine model, only two human vaccines are under current clinical trials (2, 72, 73), with an additional one in development for clinical assays (74). Although the immunogenicity of the whole sequences of NH36 and the sterol 24 c methyl transferase has been proved for humans, either independently or in combination, and a Phase I clinical trial was developed for the Leish F3 antigen, which is composed of both proteins adjuvanted by Glucopyranosil lipid A (GLA-SE) (72) no reports of Phase II or Phase III trials of this vaccine are available. A clinical trial was also performed for the ChAd63-KH chimpanzee adenovirus codifying the KMP-11 and HASPB protein, but for immunotherapy of post dermal Kala-azar patients of Sudan. It promoted a high clinical improvement in only 30%, and partial improvement in another 21%, of the patients (73). In addition, a new vaccine composed of the KMP11, LJM11 and Leish F3 (NH-SMT) antigens expressed in an Influenza virosome and adjuvanted by GLA-SE (MuLeVaClin) also showed promising immunogenic results, but was abandoned due to its complexity and high production costs (75).

The geographical overlap of the CL and VL epidemics should be used as a guide for the development of vaccines based on conserved antigens of the *Leishmania* genus that could exert cross-protective efficacy against both infections (29–33, 36, 76). Aiming to develop such a bivalent formulation, we previously demonstrated that vaccination with the conserved NH36 antigen (29) and saponin protected mice (30) and dogs (31) from VL and mice against CL (32, 33, 36). In this investigation, we showed that the conservancy of the identified NH36 epitopes among all the *Leishmania* genomes included in Blastp is extremely high, ranging from 99.4% to 99.6% in the agents of VL, and from 84% to 99.7% among the agents of CL. Other multiepitope vaccines have been proposed, but they use epitopes conserved in fewer *Leishmania* genomes (77, 78) or with lower conservancy and World coverage values (79). The overall high level of epitope identity in the *Leishmania* genus supports the use of NH36 multiepitope vaccines in generation of universal protection against both VL and CL.

Optimization of vaccine efficacy can be obtained by vaccinating with the most immunogenic domains of an antigen that concentrate important epitopes (53).Vaccination of mice and dogs with the most immunogenic NH36 moieties increased protection from 37-79% (30, 76) to 93-99% (30, 36). Furthermore, the combination of immunogenic domains in recombinant chimeras *in tandem* composed of the F1 and F3 domains increased protection significantly above the levels achieved by vaccination with each domain individually (34, 35).

Multiepitope vaccines allow a further improvement in vaccine efficacy, by combining in one formulation epitopes of diverse proteins of a species or of diverse species of a genus. Although many candidate epitopes composing multiepitope vaccines against VL were identified by immunoinfomatics (33 manuscripts retrieved by Pubmed) very few studies confirmed the immunogenic properties of *in silico* predicted epitopes *in vitro*, in either mouse (80) or hamster models (81), or in human cells (12, 77, 82, 83). A multiepitope vaccine based on the PdSP15 and LJL143 *Phlebotomus* salivary proteins, proposed as vaccine candidates against CL and VL, respectively (84), was obtained by combination of immunogenic regions that contained (1) both MHC-I and MHC-II restricted epitopes for diverse human alleles to increase population coverage; (2) conserved epitopes to increase cross-protective potential; and (3) both murine MHC-I and MHC-II restricted epitopes to increase the capability of translation of pre-clinical to the clinical studies. However, this vaccine was not tested on human cells. Furthermore, the multiepitope LeiSp vaccine enhanced the expression of TNF-α and IL-12, reduced expression of TGF-β and IL-10, increased IFN-γ and reduced IL-10 secretion into cell supernatants of human infected PBMC (82) and the multiepitope amastigote CTMI vaccine also induced high IFN-γ and low IL-10 secretion levels from human PBMCs of healthy subjects and VL-treated patients (83).

In our investigation with NH36, prediction and *in vitro* confirmation of the cross-protective potential of the murine MHC-I and MHC-II restricted epitopes of NH36 on CL (34–36) and VL (30) was performed in previous studies. The highlight achievement of this investigation was the identification of a set of human T cell epitopes which (1) bind with high promiscuity and high affinity to the most frequent DRB1, DRB3/4/5, DQA1, DQB1, DPA1 and DPB1 class II and the HLA-A, -B and -C class I alleles, (2) are highly conserved in the NHs of all species of the *Leishmania* genus, (3) could induce bivalent cross-protection against the CL and VL infections, (4) can increase population coverage to the HLA class I and II most frequent molecules of all World populations, (5) bind to the alleles predicted as associated to VL resistance, intermediate and high risk and (6) have their immunogenicity confirmed by their *in vitro* interaction with sera and PBMCs of VL cured and asymptomatic DTH^+^ infected subjects, in which they promote a Th1 or mixed cytokine production. Based on this evidence, the described set of epitopes stood up as useful in universal vaccination against human leishmaniasis. Accordingly, two multiepitope vaccines, MultiAAA and MultiGPGPG, were designed.

Epitopes that could promote the enhancement of frequencies of multifunctional triple-producers of IL-2, TNF-α and IFN-γ, are associated with generation of vaccine induced-memory (62). Asymptomatic DTH^+^ individuals showed the highest frequencies of CD4^+^ and CD8^+^ multifunctional IL-2^+^TNF-α^+^IFN-γ^+^-secreting T lymphocytes in response to 3-2018, 9-2018, 11-2018 and 15-2018 epitopes, which are probably related to the generation of immunological memory to the NH36 antigen (12, 62) and therefore, eligible as vaccine components. In addition, CD4^+^ and CD8^+^ lymphocyte double-producers of TNF-α and IFN-γ had their frequencies also increased in DTH^+^ individuals, mainly in response to the same epitopes, which suggests, that they are not only important in generation of immune protection but also correlated to VL cure and recovery of the immune response (59). This is a second very desired immunogenic property of vaccine candidate epitopes. The SLA control induced lower responses than the epitopes, except for CD4^+^ and CD8^+^ double-producers of TNF-α^+^ IFN-γ^+^ of cured individuals (59). In addition, indicating a combined Th1 and regulatory response, epitope 9 was the most potent inducer of TNF-α and epitope 11 promoted simultaneous secretion of IL-1β and IL-6 that was previously detected in response to the NH36 domains (12). Of note, although predicted and detected as ligands of HLA-class II molecules, the NH36 synthetic epitopes were also recognized by serum antibodies of cured or asymptomatic LV patients. High proportions of epitopes (25%-67%) promoted significantly higher responses in cured and DTH^+^ subjects than in controls, in IgG1 and IgG3 antibodies, which were related to disease (85, 86), in the IgG2 subclass, associated to cure (87) and the IgG4 subclass associated to disease (86) or not (88), supporting the inclusion of the whole set of epitopes in a preventive vaccine.

Memory cells are often characterized by the combination of the markers CCR7 and CD45RA (59, 61, 89, 90). Before being exposed to an antigen, cells of the host are called naive (CCR7^+^ CD45RA^+^) and are circulating between blood and secondary lymphoid organs. After antigenic stimulation, they become differentiated into central memory cells (CCR7^+^ CD45RA^-^), effector memory (CCR7^-^ CD45RA^-^) and terminally differentiated effector memory (CCR7^-^ CD45RA^+^), which show different patterns of gene expression, cytokines and effectiveness (60, 91). In our study, we used the CCR7 and CD45RA markers (61, 62), excluded from the analysis the naïve T cells and only considered the T cells labelled as central and effector memory lymphocytes. In contrast, other studies have characterized memory cells based on the expression of the CD27, CD62L and CD95 markers (60, 91, 92). CD62L, like CCR7, is a lymph node homing molecule highly expressed on central memory cell, while the CD95 and CD27 markers characterize a subpopulation of memory cells with similar functions to stem cells as self-renewal and differentiation potential (91). These markers may be used in our future work with the purpose of identifying and subclassifying the different populations of memory cells.

Although an analysis of the Th17 response against the epitopes or the multiepitope vaccines was not performed in this study, we previously described that IL-17A was secreted by all groups of VL patients studied in Spain, in response to NH36, and by asymptomatic and cured CL patients from Spain, in response to its F1 domain (49). The highest NH36-specific IL-17A was observed in cured CL patients, while asymptomatic VL individuals developed the most pronounced response to F1. The potency of the NH36 and F1 antigens was higher than that of SLA control, which in spite of being composed of many *Leishmania* proteins promoted a much lower secretion of TNF-α and IL-17A (49).

Although some of the NH36 epitopes have stood out as more potent immunological memory inducers, promoting higher frequencies of multifunctional T lymphocytes, it was decided to include all epitopes in the vaccines, privileging their promiscuity and wide population coverage that will allow them to protect broader and more diverse populations, and to exercise universal protection against leishmaniasis. In fact, most of the included epitopes are promiscuous and bind to at least 25% of the 27 HLA class II molecules tested; including 6 epitopes that bind 13 or more. Binding promiscuity was also predicted for HLA class I alleles. Two epitopes were predicted nest peptides with the capacity to bind 10 alleles, and another 8 to bind 13 or more.

With respect to worldwide population coverage, 93% of individuals are expected to have at least one class I and one class II alleles that bind to epitope 11-2018, and 77% of individuals, at least one allele that bind to epitope 13-2018. This demonstrates how the panel not only collectively provides broad coverage, but how broad coverage is also achieved by multiple individual epitopes. This depth of coverage ensures, that the same HLA restricted response can be generated against multiple different epitopes, an important consideration in light of the possibility of pathogen escape. Thus, for example, if a dominant epitope for A*02:01 undergoes a mutation that allows the pathogen to evade the immune response, avoiding detection is minimized if multiple A*02:01 binding epitopes, unlikely to undergo a similar mutation at the same time, are included in the construct.

The individual HLA-typing of each patient of this investigation identified in DTH^+^ subjects alleles previously associated with protection alone, or to protection with intermediate and high risk of VL, while VL patients only exhibited alleles correlated to high risk of VL (55, 56, 93). Interestingly, all these patients had the frequencies of their CD4^+^ and CD8^+^ multifunctional IL-2^+^TNF-α^+^IFN-γ^+^ T cells increased in response to many NH36 epitopes (1 to 11) which confirms that not only the epitopes are promiscuous but also suggests, that the allele coverage is deep.

Remarkably, the human epitope 11-2018, which showed the highest affinity-binding and promiscuity to HLA-DR, DP and DQ molecules, number of HLA-class I restricted ligands and World HLA class I and class II coverage, was predicted to bind to alleles associated to protection, intermediate risk and high risk of VL, is highly conserved in the *Leishmania* genus, promoted the highest frequencies of CD4^+^IL-2^+^ and CD8^+^TNF-α^+^, CD4^+^ and CD8^+^ double-producers of TNF-α^+^IFN-γ^+^ and triple-producers of IL-2^+^TNF-α^+^IFN-γ T cells, and the strongest mixed secretion of IL-6 and IL-1β. This epitope, which belongs to the F3, C-terminal domain of NH36, includes in its sequence a 14 amino acid MHC class II epitope previously predicted for H2d haplotype of BALB/c mice (IAd and IEd alleles) (30), which also induced a mixed inflammatory and regulatory T cell response (IFN-γ, TNF-α and IL-10) in vaccinated mice challenged with *L. (V.) braziliensis* (35). Moreover, epitope 11-2018 is binding to both susceptible and protective profile-associated alleles. This implies that its use in multiepitope vaccines could promote universal protection, preventing the development of VL in both susceptible and naturally resistant individuals. Epitope 11-2018 showed an immunoprotective profile on PBMCs but together with other epitopes reacted poorly with IgG2 in sera of cured and DTH+ individuals. IgG2 deficiency has been linked to the susceptibility of some diseases, such as *Haemophilus influenzae* type b infections (94). Also, IgG2 interacts more with polyssacharides than with proteins (95). In patients of VL, IgG and IgG1 levels decrease after antimony therapy (85). In contrast, IgG2 showed upregulation. In fact, IgG1 correlated with hypergammaglobulinemy while IgG2 did not and no correlation was detected between IgG2/IgG ratios with bone marrow amastigotes, BM phagocytosis or BM PCR. In contrast IgG2, correlated to with Albumin levels, suggesting that in VL, it is associated with cure or protection (85). Although epitope 11-2018 enhances the T cell inflammatory response of PBMC in VL cured and DTH+ subjects, its lack of reactivity to IgG2 in sera might be due to the fact that it was not predicted as a B cell epitope but only as an epitope for T lymphocytes, and that it is a short linear peptide, and therefore, not a polyssacharide antigen (95, 96). In fact, predictions for B cell epitopes using the NH36 and in the 11-2018 sequences and the Elipro tool disclosed only one linear epitope sharing only in the 5 last amino acids and two discontinuous epitopes sharing 9 or 3 amino acids of the 11-2018 epitope. Furthermore, epitope 11-2018 also promotes a regulatory response and this might also affect its interaction with high levels of IgG2.A criticism has been raised against T cell epitope vaccines because of their restriction in protection to defined HLA profiles (50). This disadvantage can be circumvented by including in a multiepitope vaccine highly promiscuous HLA-class I and II epitopes of high World population coverage. This premise guided our vaccine design, which resulted in a set of identified epitopes extremely promiscuous that showed of 98-99% total world coverage. In addition, when there is a proved genetic susceptibility background to the disease (13, 55, 93), it is important to include epitopes that would protect both the susceptible and the natural resistant individuals. In fact, the set 15 epitopes of NH36 was predicted to bind to alleles associated to high risk, neutral or intermediate risk and protection to VL (13, 55, 93), which are associated with the enhancement of certain types of cytokine producing T cells.

Genetic susceptibility to VL was demonstrated to be associated to HLA-DRB1 and -DQA1 class II alleles in Brazil and India (55, 56, 93) but not to HLA-class I genes. In our investigation, and despite the small cohort of studied patients, we showed for the first time an association of the class I allele HLA-C*04:01 to risk of VL. Our *in silico* prediction disclosed 2 nested sequences in the 3-2018 epitope, and another nested sequence in the 9-2018 epitope for the HLA-C*04:01 allele. The HLA-C*04 allele group was only recently described as associated to increased risk of American CL in a study including 186 patients and 278 controls of South Brazil (97).

Of note, although detected as ligands of HLA-class II molecules, the NH36 synthetic epitopes induced the cytotoxic response and were recognized by serum antibodies of cured or asymptomatic LV patients.

Considering the strong antigenicity of the NH36 epitopes and their ability to bind to four subclasses of IgG antibodies, induce cytokine production by CD4^+^ and CD8^+^ T lymphocytes, promote increased frequencies of central and effector memory lymphocytes, and their promiscuity predicted *in silico* and proved *in vitro*, their broad population coverage of class II and class I HLA molecules, strong predicted binding to alleles associated with protective responses, high risk and intermediate risk against human VL, and their extremely high conservation in the *Leishmania* genus (12, 29), we then included all studied epitopes, into two multiepitope formulations.

The different types of arrangements that the epitopes can undergo within a multiepitope vaccine can affect their potencies. For example, they could be less efficient if generating junctional epitopes (65). To avoid this problem and promote a more efficient class I and class II presentation, we used the AAA (64) and GPGPG (65) spacers, respectively, and obtained two proteins of good predicted immunogenic response, 87% of amino acids in favored position, good agreement with usual protein models and good estimated half-life in *E. coli*, yeast and reticulocytes. The GPGPG spacers have been widely used to restore the Th1 response (65, 66, 98) whereas the AAA and AAY linkers were mostly employed to preserve the correct induction of cytotoxic response (67, 98). Moreover, Alanine-based spacers resulted in better peptide presentation to HLA-class I than the typically used GGGS linker (64). For AIM we used the CD4-CD69 and CD40L markers for CD4^+^ and the CD69 and 4-1BB (CD137) markers for CD8^+^ T cells, which are validated combinations for detection and phenotyping of antigen-specific cells (60, 99). In addition, T cells CD40L^+^ are expected to produce abundant amounts of IL-2, TNF and IFN-γ (99).

As a proof of concept, and in agreement with all our predictions for the NH36 epitopes, both MultiAAA and MultiGPGPG recombinant proteins were capable of inducing the AIM and cytokine-producing T cell responses of cured and DTH^+^ patients. However, a further clinical study of the immunogenicity of MultiAAA and MultiGPGPG antigens should be performed in larger populations, in the future.

Regarding the percent of cells with one, two or three functions defined by intracellular staining and as expected for the use of GPGPG spacers in multiepitope vaccines (65), the MultiGPGPG construct induced the most potent differentiation from single to double- and triple-cytokine producers CD4 T cells from cured and DTH^+^ subjects, indicating that it promotes more efficiently the advancement of the Th1 response towards the generation of vaccine induced memory-T cells (59, 62). MultiGPGPG also promoted the differentiation from single to double-cytokine producers CD8 T cells from cured individuals, indicating that it also efficiently triggers the vaccine induced cytotoxic immunity. On the other hand and as expected for the use of AAA spacers (64, 66, 98), the MultiAAA protein promoted the strongest differentiation from single to double-cytokine producers CD8^+^ T cells only in DTH^+^ subjects.

On the other hand, the MultiAAA construct promoted the highest total frequencies of IL-2. TNF-α and IFN-γ-producing CD4^+^ T cells detected by ICS, higher CD4 AIM, and the highest total frequencies of IL-2- and IFN-γ-producing CD8^+^ T cells, while the MultiGPGPG protein promoted the highest CD8 AIM response and equal total frequencies of TNF-α-producing CD8 T cells.

A lack of correlation between the frequencies of cytokine-production measured by ICS and the frequencies of AIM results is expected in clinical vaccine trials and can suggest that AIM test can disclose novel information on the antigen-specific T cell response that is usually not detected by cytokine-production assays which are limited by pre-definition of the cytokines to be analyzed (100).

Our results indicate that the MultiAAA and MultiGPGPG multiepitope proteins could be potentially used in future universal vaccines against human leishmaniasis.

## Data Availability

The original contributions presented in the study are included in the article/**Supplementary Material**. Further inquiries can be directed to the corresponding author.
