# Supplementary material for: Design and development of highly conserved, HLA-promiscuous T cell multiepitope vaccines against human visceral leishmaniasis
Source: Front Immunol. 2025 Mar 31;16:1540537. doi: 10.3389/fimmu.2025.1540537 (PMC11994619; doi:10.3389/fimmu.2025.1540537)
Supplement: Supplementary file 1 [file DataSheet1.pdf]

Figure S1: Intracellular expression of the pro-inflammatory cytokines IL-2, TNF-α, IFN-γ cytokines in T lymphocytes to NH36 epitopes. Gate strategy.

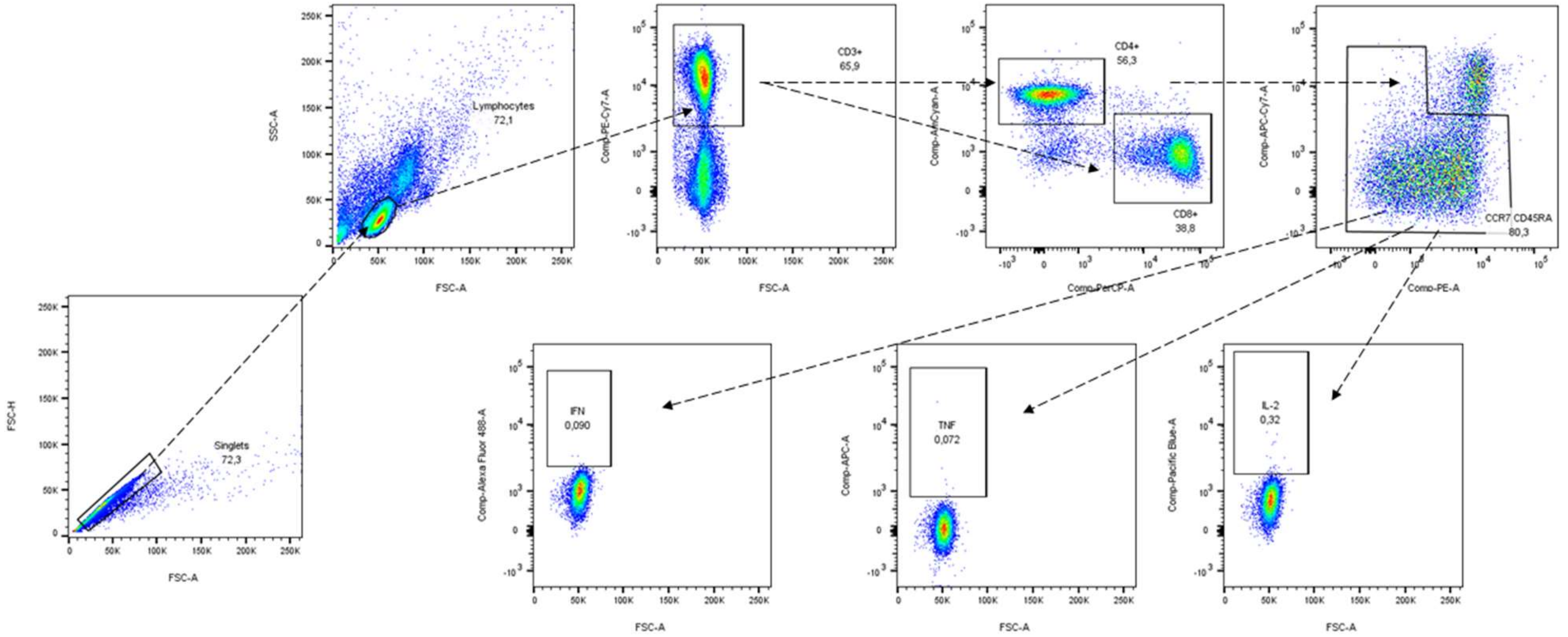

Multiple cytokine by T CD4 and CD8 cells – Panel 1

**IFN-γ**	+	+	+	-	+	-	-
**TNF-α**	+	+	-	+	-	+	-
**IL-2**	+	-	+	+	-	-	+
**Production**	**Triple**	**Double**			**Single**		

Figure S2: Activation induced marker (AIM) gate strategy in T lymphocytes, in response to MultiAAA and MultiGPGPG proteins.

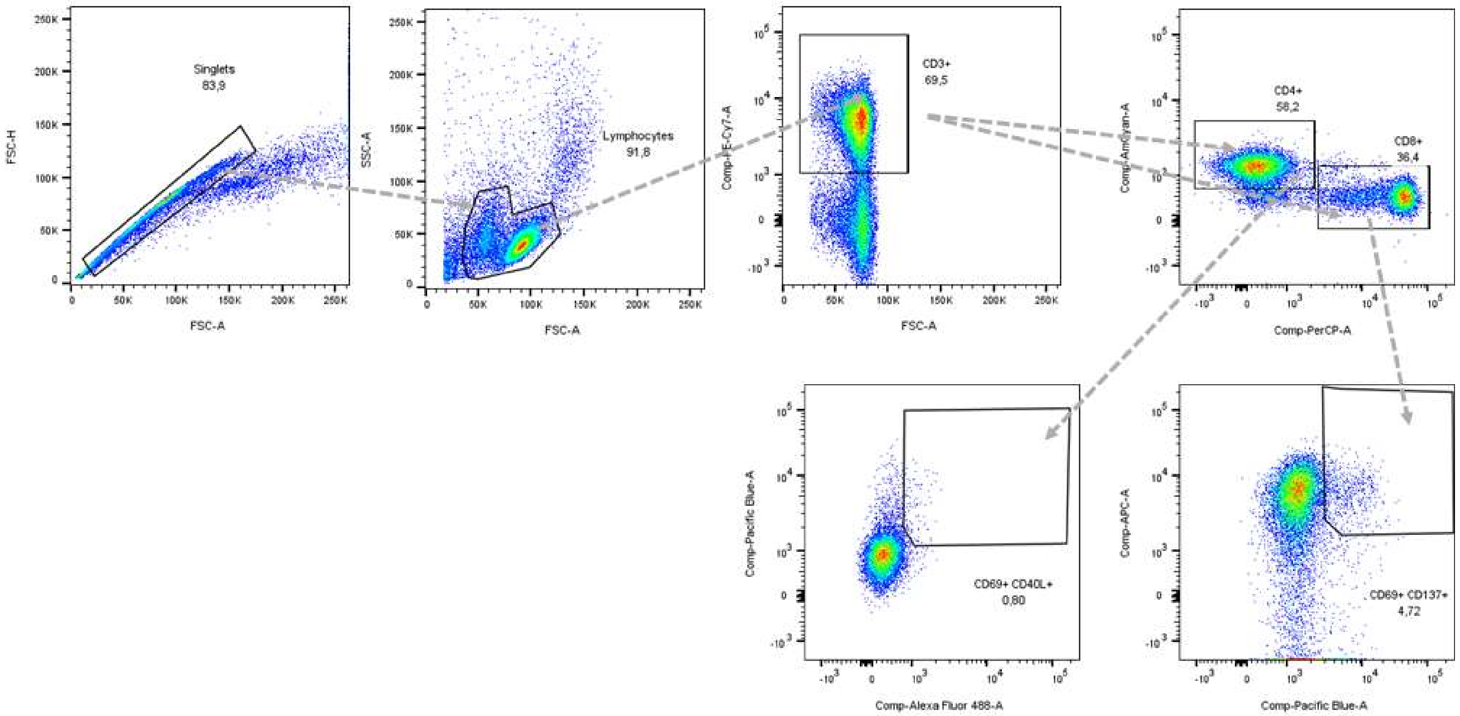

AIM $CD4^+$ and $CD8^+$ T Cells		
**CD69**	+	+
**CD40L**	+	-
**CD137**	-	+
	AIM $CD4^+$	AIM $CD8^+$

Figure S3. SDSPAGE analysis of MultiAAA and MultiGPGPG preparations.

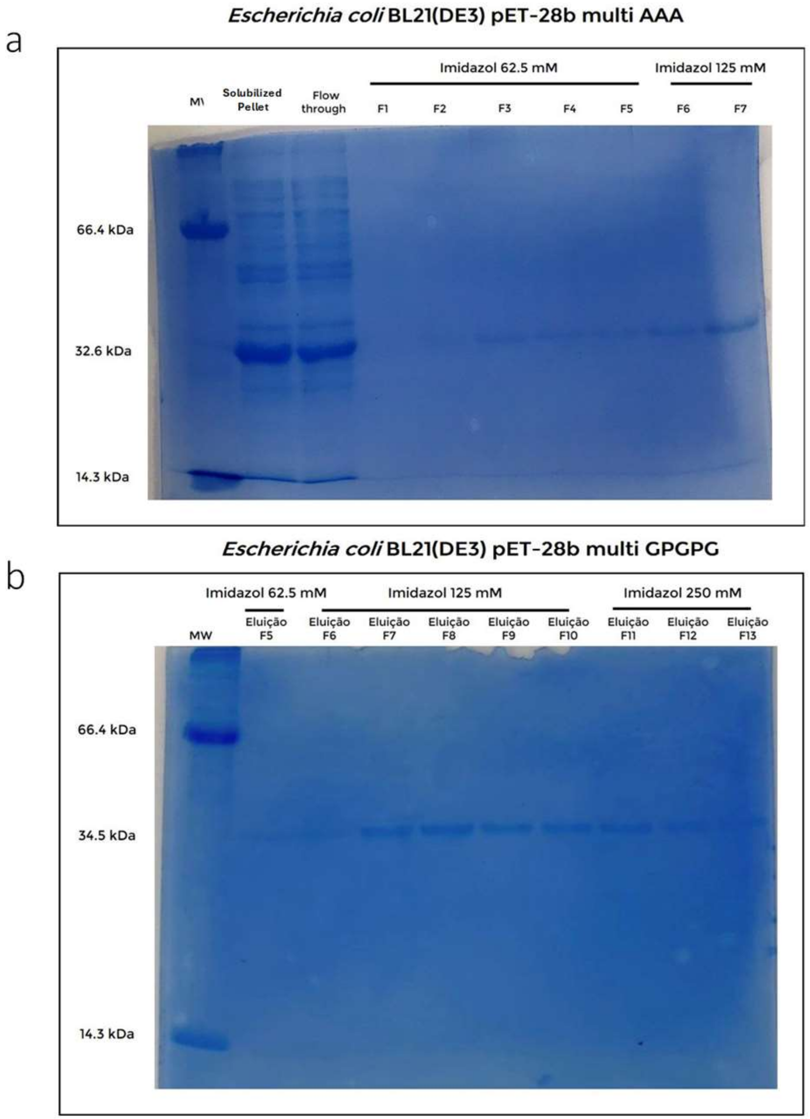

Figure S4: World Class I coverage of the epitope set

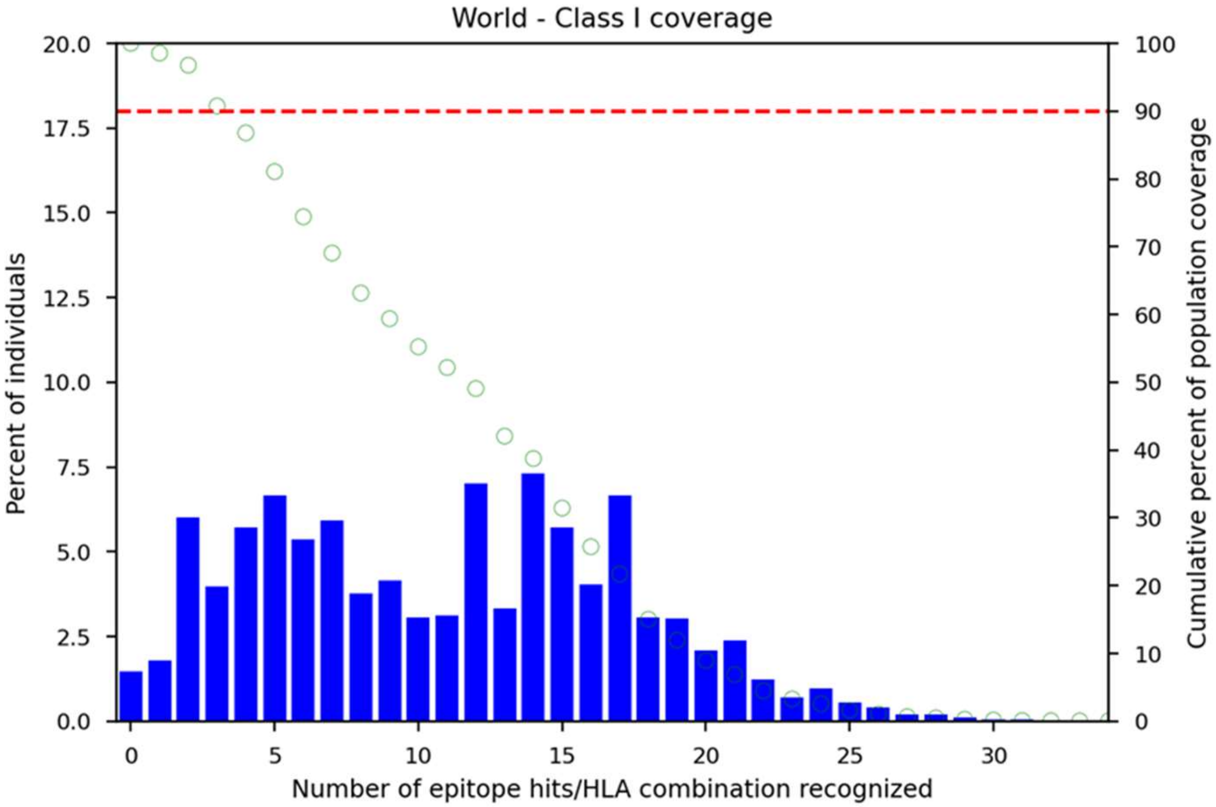

Total coverage (PF)	98.55
Avg. Hit*	10.93
PC_{90}**	3.19

Figure S1: The entire Class I panel provides coverage for > 98% of individuals, with an individual mean recognizing approximately 11 different HLA epitope/molecule combinations. Cumulatively, adding from right to left, in 90% of individuals, it is expected that at least 3.19 epitopes will be recognized (PC_{90}). Likewise, about 75% of the population would recognize 6 or more, 50% about 12 or more, etc.

Figure S5: World Class II coverage of the epitope set

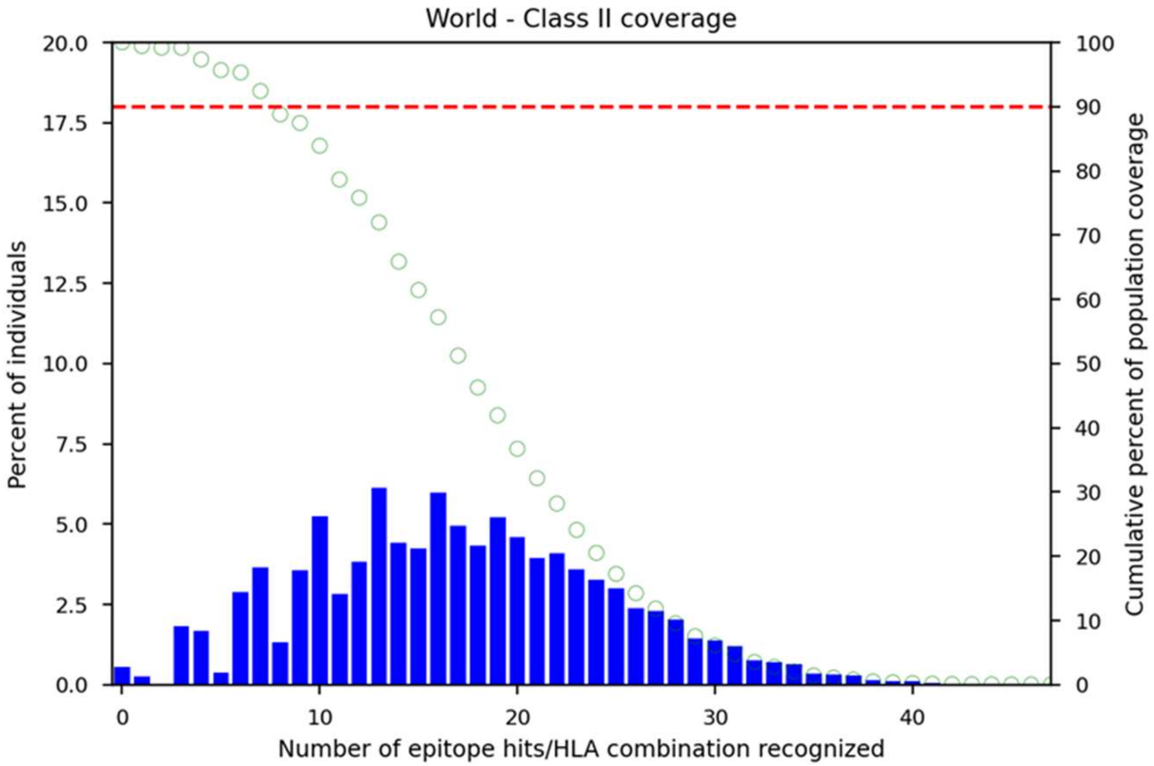

Total coverage	99.44
Avg.hit*	17.15
PC_{90}**	7.67

Figure 2S: Coverage for the prediction of class II molecules is underestimated, and only based on DRB1, DQB1 and DPB1. The DRB3/4/5 alleles have not been included. There are no reliable data on these alleles. The panel has an average expected number of epitope hits/HLA combination recognized by the population of 17.15. It is expected that at least 7.67 of the epitopes will be recognized by 90% of the world population (PC_{90}).

Figure S6. CD3 T cells stimulated with NH36 epitopes

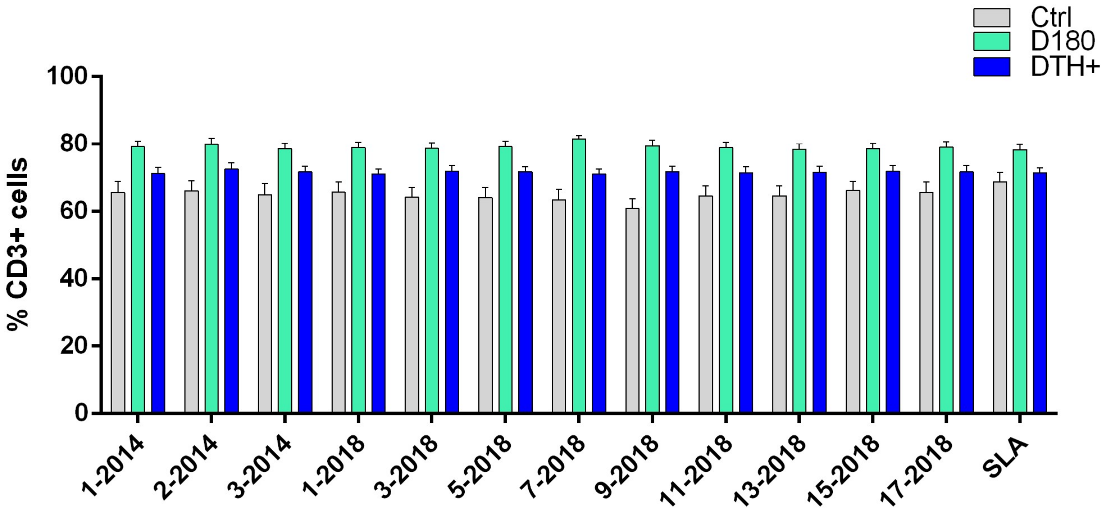

Figure S4. The percentage of CD3^{+} T lymphocytes stimulated with the NH36 epitopes 1-2014, 2-2014, 3-2014, 1-2018, 3-2018, 5-2018, 7-2018, 9-2018, 11-2018, 13-2018, 15-2018 and 17-2018 (25 µg/ml) is shown, as well as with the soluble antigen of *Leishmania (L.) donovani* (SLA) (10 µg/ml)

Figure S7. Cloning of the Multiepitope proteins in the pET28(+) expression vector

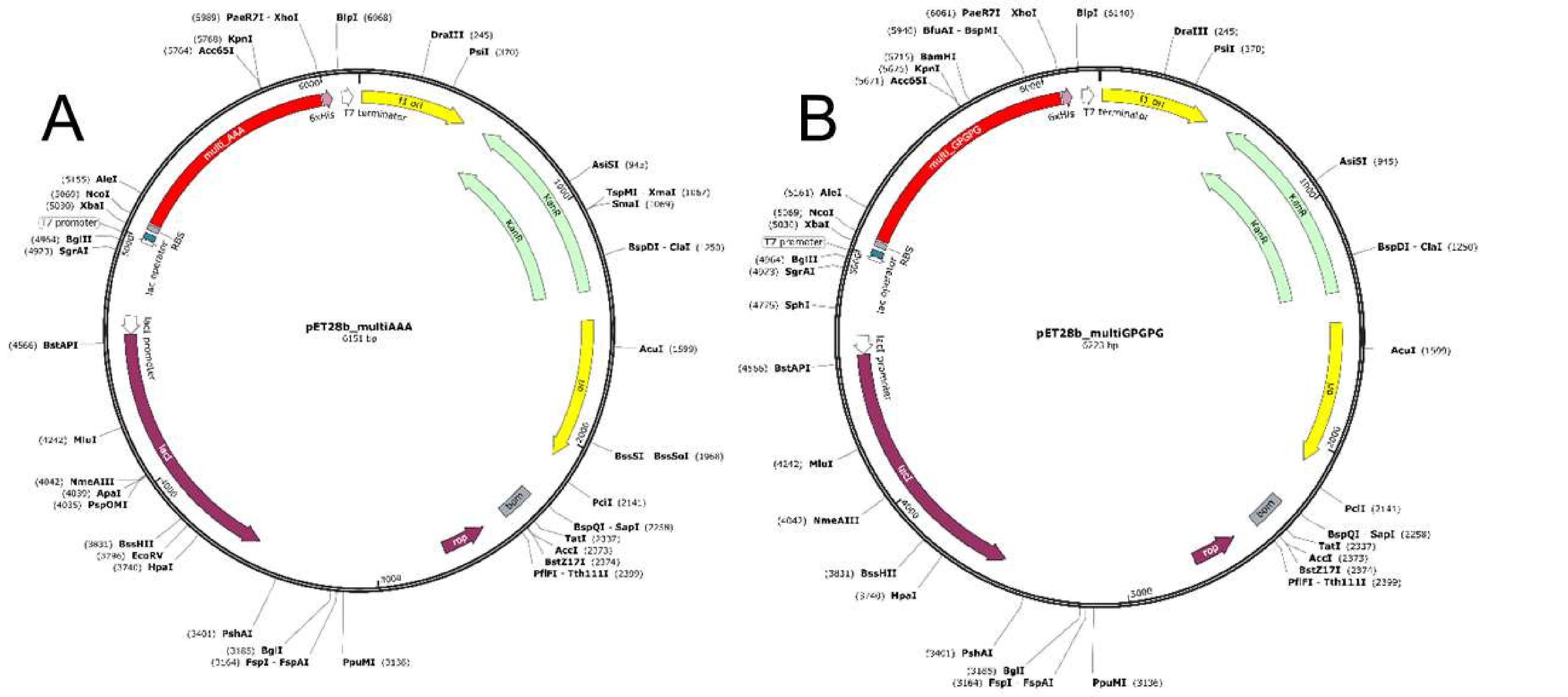

Figure S6. SnapGene (version 6.0.2) was used to integrate the adapted DNA sequence to pET-28b (+) vector, between the NcoI and XhoI restriction sites with no stop codon and a C-terminally 6 HIS-tag

Figure S8. Secondary structure of MultiAAA and MultiGPGPG proteins

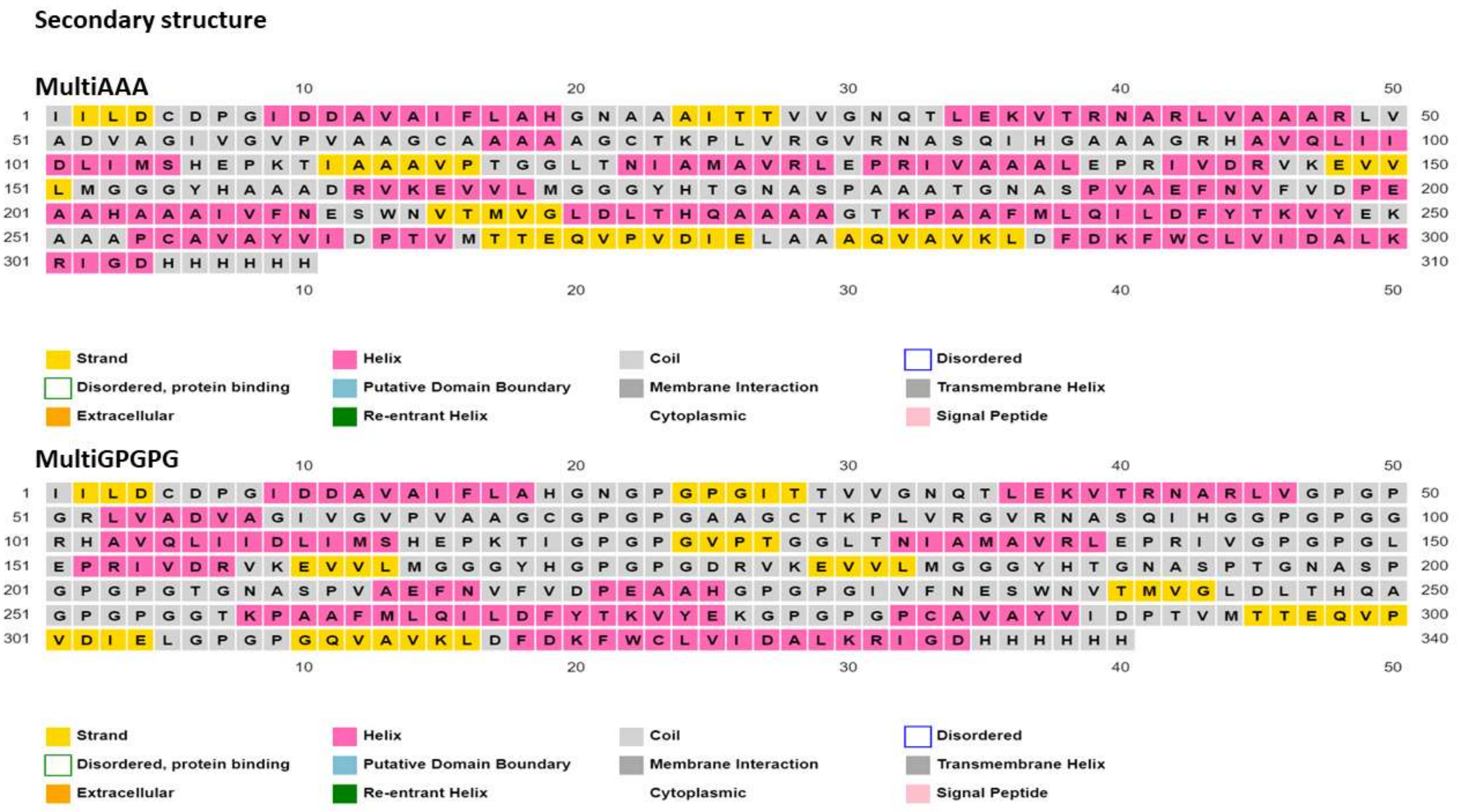

Figure S6. Secondary structures predictions of the proteins were carried out using the PSIPRED tool, which predicts also the transmembrane topology, helix, strand, coil and domain recognition

Figure S9. Prediction of the immune stimulation responses to the MultiAAA protein

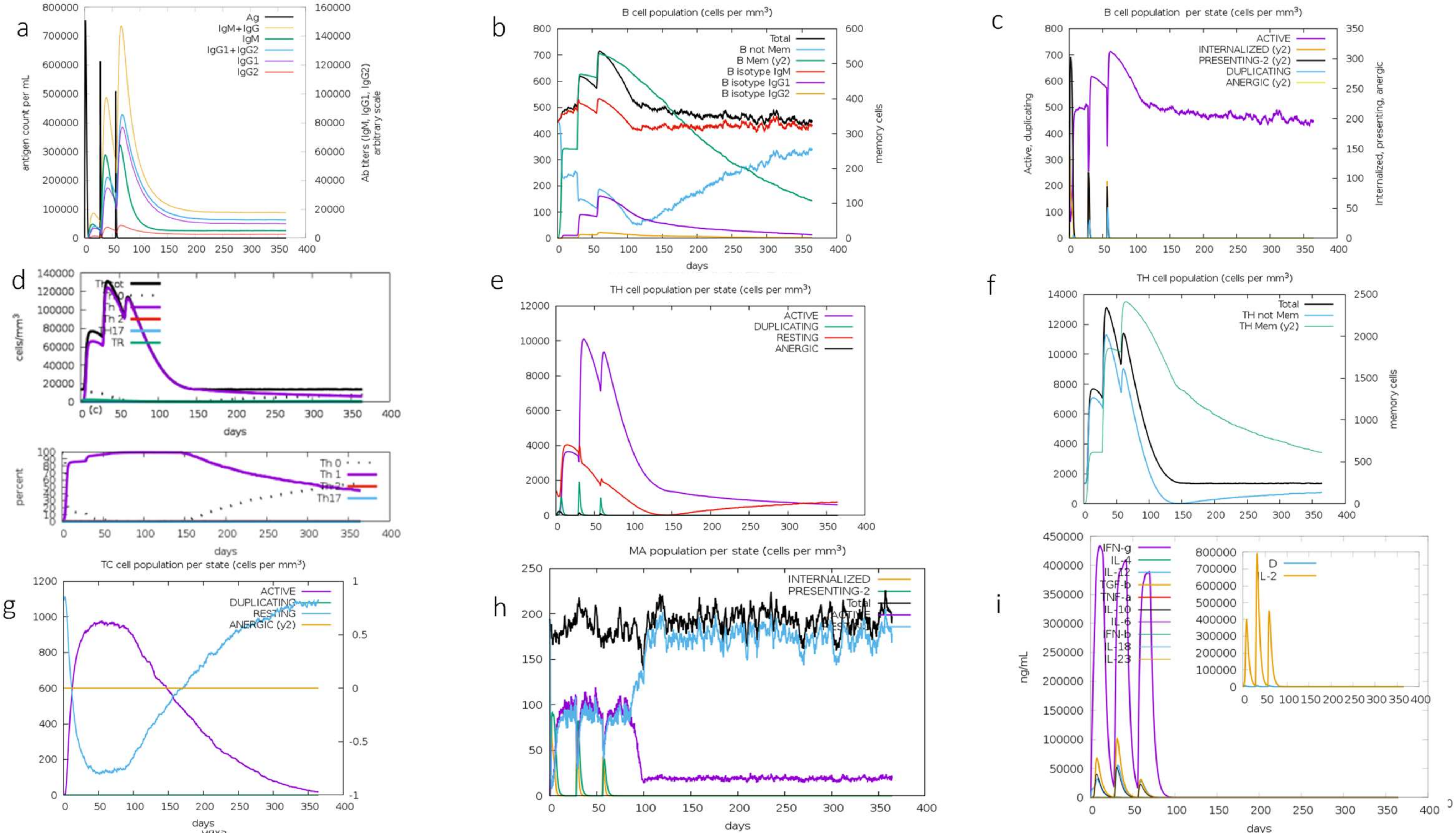

Figure S10. Prediction of the immune stimulation responses to the MultiGPGPG protein

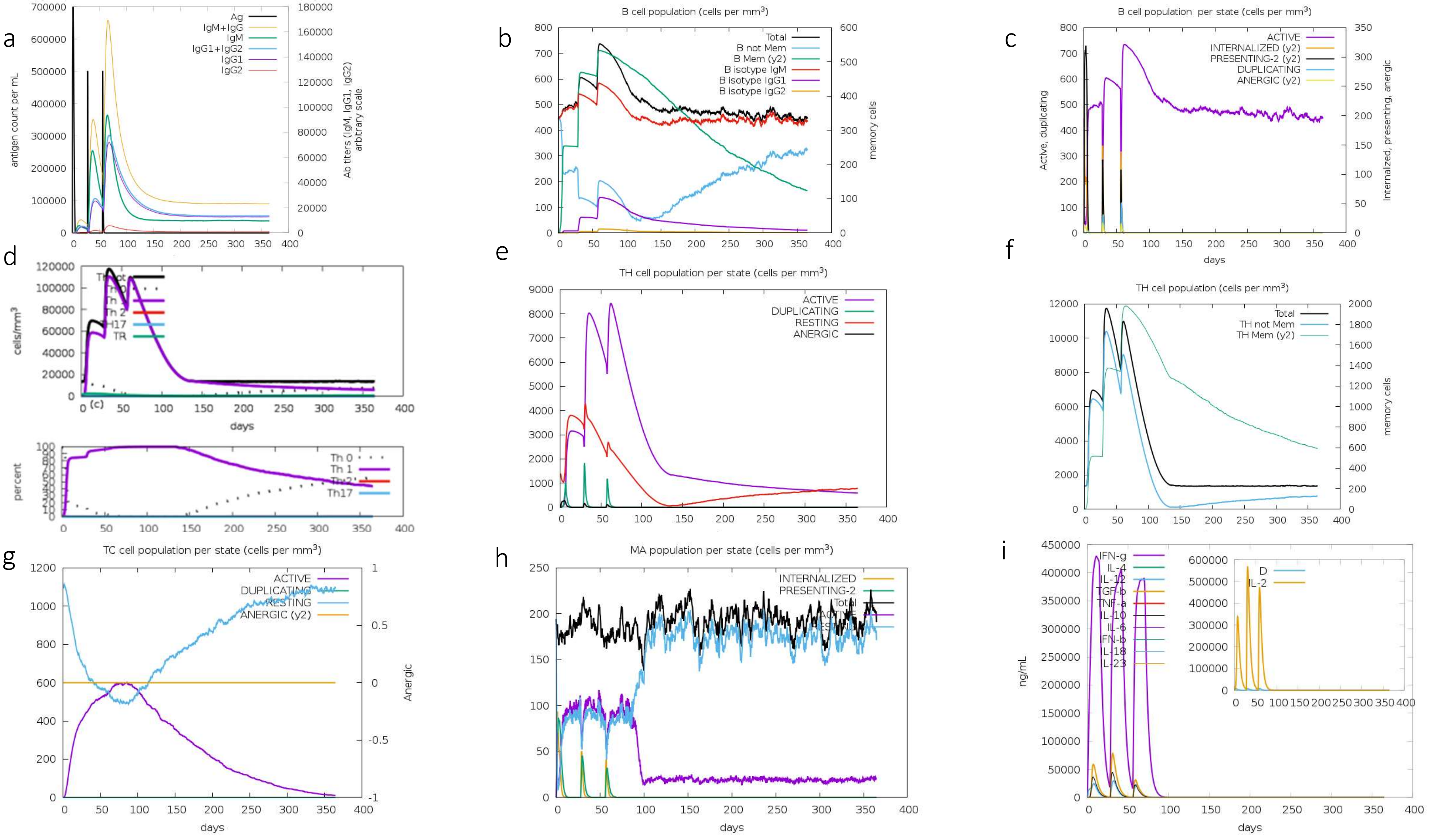

Figure S11. Prediction of the immune stimulation responses o a negative control protein.

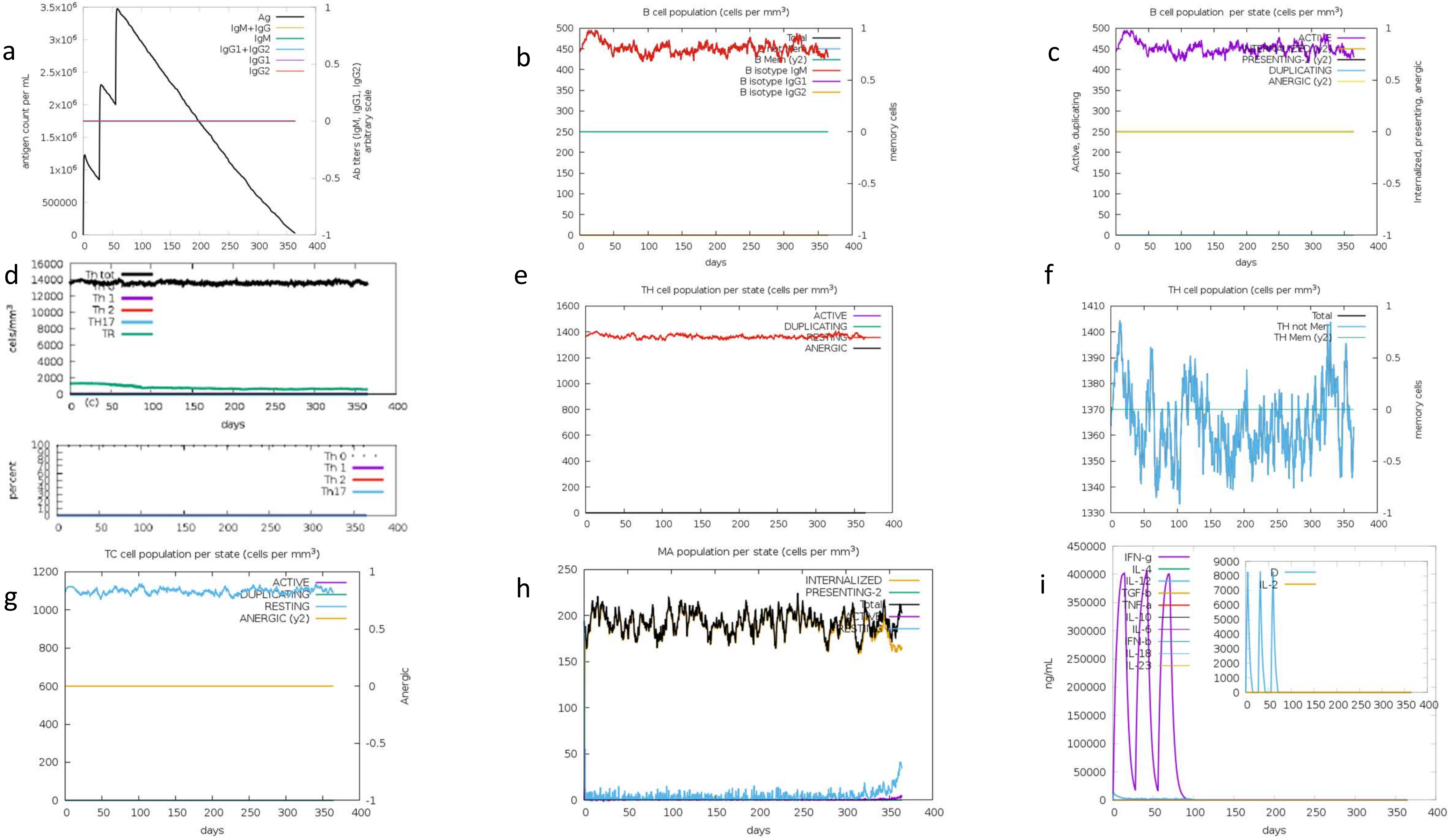

Figure S12. Prediction of the immune stimulation responses to a positive control protein.

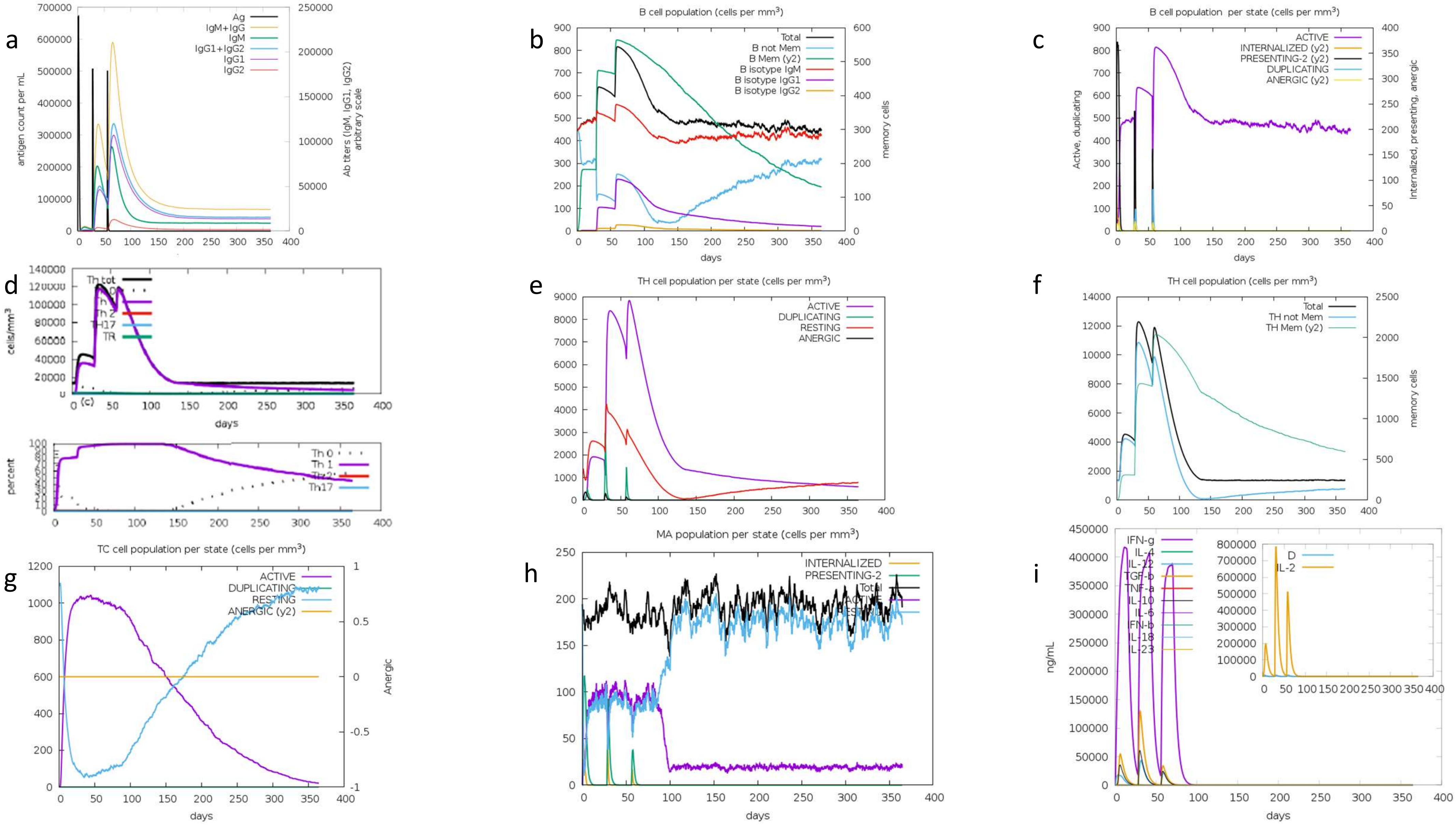

Table S1. Prediction of HLA class II molecules binding the 27 most common alleles of the DRB, DQA/DQB and DPA/DPB human genes

epitopes	PR<10%	PR<20%	DPA1*01:03/ DPB1*02:01	DPA1*01:03/ DPB1*03:01	DPA1*01:03/ DPB1*04:01	DPA1*03:01/ DPB1*04:02	DPA1*02:01/ DPB1*01:01	DPA1*02:01/ DPB1*05:01	DPA1*02:01/ DPB1*14:01	DQA1*05:01/ DQB1*02:01	DQA1*05:01/ DQB1*03:01	DQA1*03:01/ DQB1*03:02	DQA1*04:01/ DQB1*04:02	DQA1*01:01/ DQB1*05:01	DQA1*01:02/ DQB1*06:02
3 2018	**0**	6													
9 2018	**7**	13								17.00				**6.60**	10.00
11 2018	**11**	15	**7.10**		**2.80**	**9.30**	**6.60**	**1.90**		**0.08**		12.00	13.00	**3.40**	16.00
15 2018	**8**	11	**3.90**		**2.80**	**9.30**	16.00	**6.40**		**4.60**				**4.20**	
1 2014	**5**	6												**4.20**	
2 2014	**9**	17			13.00	**7.70**				**6.30**		19.00		**5.50**	
1 2018	**4**	7										**4.50**	**3.10**	15.00	**2.00**
5 2018	**7**	15							**5.20**	**8.40**	12.00	14.00	**9.50**		**4.20**
13 2018	**7**	12								**8.10**		14.00	16.00	**4.00**	
17 2018	**11**	21	**7.80**		**2.80**	**6.10**	11.00	14.00	**9.20**	**4.40**				**4.20**	
15-17-2018	**13**	21	**3.90**		**2.80**	**6.10**	11.00	**6.40**	**9.20**	**4.40**				**4.20**	
7 2018	**4**	8								**1.50**		**0.21**	**8.50**	**2.40**	
3 2014	**3**	7									**9.90**		**3.10**		12.00
BL1	**8**	11								**8.30**	**6.20**	15.00	11.00		**5.70**
BL2	**1**	5						17.00							**8.60**

epitopes	DRB1*01:01	DRB1*03:01	DRB1*04:01	DRB1*04:05	DRB1*07:01	DRB1*08:02	DRB1*09:01	DRB1*11:01	DRB1*12:01	DRB1*13:02	DRB1*15:01	DRB3*01:01	DRB3*02:02	DRB4*01:01	DRB5*01:01
3 2018		14.00			12.00	18.00		17.00					12.00	17.00	
9 2018	18.00	**9.80**	**9.20**	**9.40**	**7.00**					**1.30**	11.00	14.00	**3.20**	**7.50**	
11 2018				13.00					**2.78**		**5.90**	**6.90**		**4.10**	
15 2018				12.00								**2.50**		**2.50**	12.00
1 2014			**5.80**	**4.20**		**2.90**		**9.50**					13.00		
2 2014		**0.83**	**6.00**	13.00	16.00	19.00	11.00		**8.20**	**7.00**	19.00	**8.90**	12.00	**0.99**	
1 2018										16.00	15.00	**2.80**			
5 2018					11.00	**7.80**	13.00	15.00	13.30	11.00	15.00		**7.30**	11.00	
13 2018	16.00	**0.86**	**0.82**	**3.40**		18.00				16.00		**0.11**	**2.30**		
17 2018	13.00	14.00	14.00	12.00	16.00			**5.50**	**4.15**	**7.40**	19.00	**0.51**	17.00	18.00	**5.00**
15-17-2018	13.00	**3.90**	14.00	12.00	16.00			**5.50**	**4.15**	**7.40**	19.00	**0.51**	17.00	18.00	**0.51**
7 2018		14.00	19.00	13.00			11.00								
3 2014	19.00					17.00					**9.90**				16.00
BL1	**8.00**	**6.40**				**2.90**				**4.60**		**2.70**	12.00		
BL2						19.00					13.00	15.00			

grey and bold<10%
light grey <20%

Table S2. *In vitro* inhibition of affinity-binding of NH36 HLA-class II epitopes to the 27 most common alleles of human genes DRB*1, DRB*3, DRB*4 and DRB*5, DQA*1/DQB*1 and DPA*1 /DPB*1

epitopes	Length	Alleles bound	DPA1*01:03/ DPB1*02:01	DPA1*01:03/ DPB1*03:01	DPA1*01:03/ DPB1*04:01	DPA1*03:01/ DPB1*04:02	DPA1*02:01/ DPB1*05:01	DPA1*02:01/ DPB1*14:01	DQA1*05:01/ DQB1*02:01	DQA1*05:01/ DQB1*03:01	DQA1*03:01/ DQB1*03:02	DQA1*04:01/ DQB1*04:02	DQA1*01:01/ DQB1*05:01	DQA1*01:02/ DQB1*06:02	DRB1*01:01	DRB1*03:01	DRB1*04:01	DRB1*04:05	DRB1*07:01	DRB1*08:02	DRB1*09:01	DRB1*11:01	DRB1*12:01	DRB1*13:02	DRB1*15:01	DRB3*01:01	DRB3*02:02	DRB4*01:01	DRB5*01:01
3 2018	20	11	**238**	1070	**156**	**328**	**835**	2791	29993	-	-	-	8129	8189	**740**	4008	1194	19720	**97**	**449**	7551	**943**	-	**87**	1187	20500	6350	**205**	**373**
9 2018	20	7	2042	7480	3874	2672	3324	8839	**190**	-	**461**	4350	**10**	**393**	**851**	-	1175	6255	**260**	-	2539	-	-	2163	1144	17170	19327	**10**	5901
11 2018	21	13	**393**	9002	**644**	**819**	**0.058**	1445	**163**	1991	**219**	2100	**85**	**954**	**243**	4064	1569	2853	3152	6132	1922	8048	29425	8347	**184**	**451**	19329	**0.29**	**792**
15 2018	20	5	**822**	-	**993**	2582	**10**	13437	**32**	-	2671	9800	3539	2688	8487	-	12116	-	14012	-	9778	-	-	28557	5150	12885	-	**54**	-
1 2014	20	13	25482	**107**	17200	-	2167	**193**	8457	28658	27887	-	**632**	**141**	1033	-	**77**	**115**	**65**	**19**	**300**	**96**	-	2854	**82**	-	6745	**43**	**373**
2 2014	20	17	**289**	**568**	1085	**528**	**16**	1866	**267**	22353	20195	-	**51**	**783**	1627	2038	**128**	**545**	**112**	**469**	**43**	3331	6299	**343**	**252**	**234**	10270	**15**	**37**
1 2018	21	8	-	-	-	15801	33356	33356	**145**	21726	**85**	**546**	2727	**34**	**539**	9590	8278	10257	6423	24852	**748**	10495	-	5001	**188**	1883	-	**407**	6111
5 2018	20	13	6670	**476**	7905	3150	3587	905	**303**	**147**	3332	2790	1487	**72**	**8.2**	12876	1534	18419	**18**	**842**	**490**	**249**	-	**185**	**592**	-	19075	**73**	1299
13 2018	24	13	26459	26973	29186	29186	28411	18016	**5.5**	7114	**60**	**151**	**55**	1630	**87**	**339**	**125**	**100**	**378**	6943	2813	4115	21612	5724	**316**	**45**	**912**	**62**	5157
17 2018	20	2	16485	-	-	24615	8033	13659	**957**	-	11592	7880	2435	16398	7979	29881	9949	-	9962	-	25050	-	-	3275	12099	**425**	-	2843	2308
7 2018	20	7	4857	-	28927	-	5838	35023	**65**	11154	**252**	**335**	**68**	8562	**990**	1933	1084	8137	-	12295	23889	-	-	23617	**238**	16577	11508	**412**	-
3 2014	20	7	22798	4359	-	-	**263**	20570	5000	**799**	7140	8090	24024	**691**	**789**	-	7992	2965	15214	2985	5907	9932	-	-	**214**	-	5572	**332**	**150**
BL1	18	13	5103	1577	8818	8259	10375	**613**	**70**	**104**	**180**	**202**	1029	**123**	**23**	-	5537	1686	**461**	2308	4101	-	21261	**36**	**240**	**123**	**776**	**60**	3380
BL2	20	7	7625	**656**	28756	-	**24**	**194**	**319**	13193	10100	15700	19368	**37**	1170	-	10204	2957	2200	7122	20484	20375	10726	8469	**262**	27892	24139	**67**	2011

Table S3. HLA Class I binding sequences in the NH36 Class II epitopes

Epitope	unique nested epitopes	epitope/HLA combinations	total promiscuous epitopes	promiscuous epitopes binding to 2 alleles	promiscuous epitopes binding to 3 alleles	promiscuous epitopes binding to 4 alleles	promiscuous epitopes binding to 5 alleles	promiscuous epitopes binding to 6 alleles	promiscuous epitopes binding to 7 alleles	promiscuous epitopes binding to 8 alleles
1 2014	**3**	**3**	**0**							
2 2014	**5**	**10**	**4**	**3**	**1**					
3 2014	**4**	**5**	**1**	**1**						
1 2018	**6**	**2**	**4**	**3**	**1**					
3 2018	**4**	**9**	**4**	**1**	**3**					
5 2018	**6**	**13**	**4**	**1**	**3**					
7 2018	**6**	**16**	**4**	**1**	**1**	**1**	**1**			
9 2018	**6**	**15**	**2**			**1**			**1**	
11 2018	**10**	**33**	**9**	**3**	**2**	**3**		**1**		
13 2018	**15**	**37**	**10**	**4**	**3**	**1**	**2**			
15 2018	**5**	**10**	**4**	**1**	**2**	**1**				
17 2018	**3**	**9**	**2**			**2**				
15-17 2018	**8**	**20**	**8**	**2**	**2**	**4**				
BL1	**7**	**14**	**4**	**2**	**2**					
BL2	**9**	**19**	**4**	**3**						**1**

Epitope	HLA-A*01:01	HLA-A*02:01	HLA-A*02:03	HLA-A*02:06	HLA-A*03:01	HLA-A*11:01	HLA-A*23:01	HLA-A*24:02	HLA-A*26:01	HLA-A*30:01	HLA-A*30:02	HLA-A*31:01	HLA-A*32:01	HLA-A*33:01	HLA-A*68:01	HLA-A*68:02
1 2014												2				1
2 2014			1		1				1						1	1
3 2014		1							1	1						
1 2018									1							
3 2018		2		1												
5 2018		1	1	1							1	2		2	1	
7 2018		1		1					1							4
9 2018		1	1	1							1					3
11 2018	3	1	1	2		1	1	1	1	1	4		2		1	1
13 2018		2	2	6			1	2	2							6
15 2018		1	1	1									2			
17 2018	1	1	1	2									1			1
15-17 2018		1	2	2					1				3			1
BL1		1	3	2												2
BL2		1	2	1					1	2			1			

Epitope	HLA-B*07:02	HLA-B*08:01	HLA-B*15:01	HLA-B*35:01	HLA-B*40:01	HLA-B*44:02	HLA-B*44:03	HLA-B*51:01	HLA-B*53:01	HLA-B*57:01	HLA-B*58:01
1 2014											
2 2014				1							
3 2014						1	1				
1 2018				1							
3 2018								1			
5 2018	1							2	1		
7 2018	2			2				2	2		
9 2018					1						
11 2018	2		3	1				1	1	1	
13 2018					2	1	2	1			
15 2018										2	2
17 2018		1						1			
15-17 2018		1	1					1		2	2
BL1											
BL2		2			1	1	1				

Epitope	HLA-C*03:03	HLA-C*04:01	HLA-C*05:01	HLA-C*06:02	HLA-C*07:01	HLA-C*07:02	HLA-C*08:02	HLA-C*12:03	HLA-C*14:02	HLA-C*15:02
1 2014										
2 2014	1			1	1			1		
3 2014										
1 2018										
3 2018		2	2				1			
5 2018										
7 2018								1		
9 2018		1		1	1	1	1	1		1
11 2018			1				1		1	1
13 2018	2		2			1	1	2	1	1
15 2018							1			
17 2018										
15-17 2018						1		1	1	
BL1			3					2		1
BL2			1	3			1	1		

Table S4. Analysis of the worldwide population coverage of epitopes for HLA class I and class II alleles

**Epitope**	**Class I Population coverage (%)**	**Class II Population coverage (%)**
1-2014	7.8	75.7
2-2014	36.1	91.3
3-2014	53.8	68.4
1-2018	53.6	71.5
3-2018	42.2	85.5
5-2018	63.9	87.7
7-2018	62.5	70.3
9-2018	50.9	72.1
11-2018	93.9	92.7
13-2018	77.8	85.9
15-2018	61.9	71.1
17-2018	56.1	23
15 and 17 2018	66.4	71.13
BL1	43.3	89.1
BL2	66.5	65.3

Table S5. epitopes of NH36 connecting the DRB1 alleles associated with risk and protection against LV (Singh T. et al., 2018)

**Protection**				**Neutral**				**High risk**					
**DRB1*01:01**	**DRB1*15:01**	**DRB1*15:02**	**DRB1*16:02**	**DRB1*03:01**	**DRB1*04:01 DRB1*04:05**	**DRB1*07:01**	**DRB1*12:02**	**DRB1*09:01**	**DRB1*10:01**	**DRB1*11:01**	**DRB1*13:01**	**DRB1*13:02**	**DRB1*14:04**
11-2018	11-2018		ND	13-2018									ND
					1-2014	3-2018		1-2014		3-2018		3-2018	
1-2018	1-2018				2-2014	9-2018		2-2014		1-2014		2-2014	
5-2018	5-2018				13-2018	1-2014		1-2018		5-2018		5-2018	
13-2018	13-2018					2-2014		5-2018				BL1	
7-2018	7-2018					5-2018							
3-2018	3-2014					13-2018							
BL1	BL1					BL1							
	1-2014												
	2-2014												
9-2018													
3-2014	BL2												

Table S6. NH36 epitopes linking to alleles associated with risk and protection against VL (LEISHGEN et al., 2013)

Alleles associated with PROTECTION		Alleles associated with INTERMEDIATE RISK	Alleles associated with HIGH RISK
DRB1*15:01	DRB1*01:01	DQB1*05:01	DQB1*02:01
11-2018	11-2018	11-2018	11-2018
1-2018	1-2018		1-2018
5-2018	5-2018		5-2018
13-2018	13-2018	13-2018	13-2018
7-2018	7-2018	7-2018	7-2018
3-2018	3-2018		
BL1	BL1		BL1
1-2014		1-2014	
2-2014		2-2014	2-2014
	9-2018	9-2018	9-2018
BL2	3-2014		BL2
			15-2018
			17-2018

Table S7. HLA typing of VL patients

Codes	Patients	*HLA-A**		*HLA-B**		*HLA-C**		*DRB1**		*DQB1**		*DRB3**		*DRB4**		*DRB5**		*DQA1**		*DPA1**		*DPB1**	
B:Y1	Cured	02:05	68:01	15:20	58:01	04:01	07:18	13:02	14:02	03:01	06:09	01:01	03:01					01:02	05:03	01:03	01:03	04:02	104:01
B:Y2	Cured	24:03	74:01	15:01	35:01	03:03	04:01	08:04	12:01	04:02	05:01	01:01						01:05	04:01	01:03	01:03	04:01	04:02
B:Y4	Cured	24:02	34:02	15:01	44:03	03:03	04:01	11:03	15:03	03:01	06:02	02:02				01:01		01:02	05:05	01:03	03:02	03:01	40:01
B:Y6	Cured	23:01	68:01	15:03	44:03	02:10	04:01	07:01	10:01	02:02	05:01			01:01				01:05	02:01	01:03	02:01	04:01	17:01
B:Y7	Cured	01:01	30:01	45:01	81:01	16:01	18:01	01:02	11:01	03:19	05:01	02:02						01:01	05:05	02:01	02:02	01:01	01:01
B:Y8	Cured	24:02	68:02	27:05	53:01	01:02	04:01	08:01	13:03	03:01	04:02	01:01						04:01	05:05	01:03	02:01	04:02	17:01
B:Y9	Cured	02:05	02:05	44:03	49:01	04:01	07:01	03:01	04:05	02:01	03:02	02:02		01:03				03:03	05:01	01:03	02:02	01:01	02:01
B:Y10	Cured	02:20	23:01	07:02	14:02	07:18	08:02	07:01	13:01	02:01	06:03	03:01		01:01				01:03	02:01	02:01	03:01	01:01	105:01
C:Y1	DTH+	24:02	24:02	15:01	18:01	03:03	07:01	04:11	15:01	03:02	06:02			01:03		01:01		01:02	03:01	02:01	03:01	14:01	105:01
C:Y2	DTH+	24:02	30:02	45:01	50:02	06:02	16:01	04:06	09:01	02:02	04:02			01:01	01:03			03:03	03:03	01:03	01:03	04:01	04:01
C:Y3	DTH+	24:02	24:02	13:02	15:01	03:03	06:02	07:01	11:03	02:02	03:01	02:02		01:03				02:01	05:05	01:03	01:03	03:01	04:02
C:Y4	DTH+	32:01	74:01	41:01	41:02	02:10	17:01	11:01	13:03	03:01	03:01	01:01	02:02					05:05	05:05	01:03	02:01	04:01	10:01
C:Y5	DTH+	03:01	11:01	18:01	51:01	02:02	15:02	01:01	10:01	05:01	05:01							01:01	01:05	02:01	03:01	105:0	17:01
C:Y6	DTH+	24:02	30:01	13:02	44:02	02:02	06:02	04:01	07:01	02:01	03:01			01:01				02:01	03:03	01:03	01:03	04:01	04:01
C:Y7	DTH+	11:01	24:02	27:05	51:01	01:02	15:02	01:01	08:01	04:02	05:01							01:01	04:01	01:03	03:01	04:02	105:01
C:Y8	DTH+	02:01	68:02	14:02	15:10	03:04	08:02	10:01	11:01	03:01	05:01	02:02						01:05	05:05	01:03	01:03	04:01	29:01
C:Y9	DTH+	25:01	32:01	27:05	39:06	02:02	07:02	01:01	04:01	03:02	05:01			01:03				01:01	03:01	01:03	01:03	04:01	04:01
C:Y10	DTH+	23:17	24:02	50:01	50:02	06:02	06:02	03:01	04:06	02:01	04:02	02:02		01:03				03:03	05:01	01:03	02:01	01:01	04:01

high risk grey 4: 13:01,13:02, DRB10:01, 11, DQB1:02:01
intermediate risk: grey 3 DQB05:01
protection: grey 1 DRB1 01:01, 15:01

Table S8: Epitopes stimulating multifunctional CD4$^+$ T cells in VL patients and asymptomatic subjects DTH$^+$ with HLA-resistant or susceptible background

Patient	Group	Allele	Susceptibility/protection	epitope stimulating the CD4+IL-2+TNF-α+ response	epitope stimulating the CD8+IL-2+TNF-α+ response
CY:1	DTH$^+$	DRB1*15:01	protection	3 2018	-
CY:4	DTH$^+$	DRB1*11:01	high risk	5,7,9,15-2018	5,7,9,17-2018
CY:5	DTH$^+$	DRB1*01:01	protection	1,3,5,7,9,11,15,17-2018	1,3,5,7,9,11,15,17-2018
		DQB1*05:01	intermediate risk		
		DRB1*10:01	high risk		
CY:7	DTH$^+$	DRB1*01:01	protection	1,2-2014,3,7,9,11,13-2018	1,2-2014,3,7,9,11,13-2018
		DQB1*05:01	intermediate risk		
CY:8	DTH$^+$	DRB1*10:01	high risk	1,2,3-2014,5,9,11,15-2018	1,2,3-2014,5,9,11,15-2018
		DRB1*11:01	high risk		
CY:9	DTH$^+$	DRB1*01:01	protection	1,2,3-2014, 3,7,9,11,13-2018	1,2,3-2014,1,3,7,9,11,13,15,17-2018
		DQB1*05:01	intermediate risk		
BY:1	cured	DRB1*13:02	high risk	2,3-2014,1,3,5,11-2018	2,3-2014,1,3,5,11-2018
BY:6	cured	DRB1*10:01	high risk	2-2014, 3,5-2018	2-2014, 3,5,9,15-2018
BY:7	cured	DRB1*11:01	high risk	3-2014, 3,5,9-2018	3-2014,3,5,9-2018
BY:9	Cured	DQB1*02:01	high risk	2-2014, 5,7,9,11,13,15,17-2018	2,2014,5,7,9,11,13,15,17-2018
BY:10	cured	DQB1*02:01	high risk	2-2014, 5, 17-2018	3 2018
		DRB1*13:01	high risk		

Table S9. Epitope conservancy analysis (percents)

Epitopes	Leishmania (L.) donovani	genomes	Leishmania (L.) infantum infantum	genomes	Leishmania (L.) Infantum chagasi	genomes	Leishmania (L.) tropica	genomes	Leishmania (L.) major	genomes	Leishmania (L.) mexicana	genomes	Leishmania (L.) tarentolae	genomes	Leishmania (V.) panamensis	genomes	Leishmania (V.) braziliensis	genomes	Leishmania (V.) guyanensis	genomes
1 2014	100.00	16	100.00	6	100.00	1	100.00	1	95.00	5	95.00	2	90.00	1	80.00	1	80.00	1	80.00	1
2 2014	100.00	16	100.00	6	100.00	1	100.00	1	100.00	5	94.74	2	89.47	1	84.21	1	84.21	1	84.21	1
3 2014	100.00	16	100.00	6	100.00	1	100.00	1	100.00	5	90.00	2	100.00	1	75.00	1	75.00	1	75.00	1
1 2018	99.41	16	100.00	6	100.00	1	100.00	1	100.00	5	95.24	2	100.00	1	90.48	1	95.00	1	90.48	1
3 2018	98.75	16	100.00	6	100.00	1	100.00	1	90.00	5	100.00	2	90.00	1	95.00	1	95.00	1	95.00	1
5 2018	100.00	16	100.00	6	100.00	1	100.00	1	100.00	5	100.00	2	95.00	1	95.00	1	83.33	2	75.00	1
7 2018	99.69	16	100.00	6	100.00	1	100.00	1	95.00	5	85.00	2	95.00	1	80.71	2	80.71	3	80.71	2
9 2018	99.69	16	94.85	6	95.00	1	100.00	1	100.00	4	100.00	2	90.00	1	90.00	2	90.00	1	90.00	1
11 2018	99.70	16	100.00	5	100.00	1	100.00	1	100.00	5	95.24	2	90.48	1	75.00	1	79.00	1	75.00	1
13 2018	98.96	16	99.17	5	100.00	1	95.83	1	95.83	5	95.83	2	83.33	1	87.50	1	87.50	1	87.50	1
15 2018	99.05	16	100.00	4	100.00	1	100.00	1	100.00	5	100.00	2	100.00	1	75.00	1	78.95	1	75.00	1
17 2018	99.52	16	100.00	4	-	-	-	1	100.00	4	90.00	1	95.00	1	84.21	1	80.00	1	84.21	1
15 17 2018	98.96	16	100.00	4	100.00	1	100.00	1	97.50	5	95.84	2	95.83	1	79.17	1	79.17	1	79.17	1
BL1	99.31	16	100.00	6	100.00	1	100.00	1	100.00	5	100.00	2	82.35	1	94.12	1	94.12	1	94.12	1
BL2	99.69	16	100.00	6	100.00	1	100.00	1	100.00	5	95.00	2	100.00	1	94.12	1	94.12	1	94.12	1
mean	99.39		99.60		99.64		99.70		98.22		95.46		93.10		85.30		85.07		83.97	

Table S10. Tertiary structure models, validation and refine:

Ramachandran	MultiAAA SM	MultiAAA SM	MultiAAA SM	MultiGPG PG SM	MultiGPG PG SM
SM*	Model 1	Model 2	Model 3	Model 1	Model 2
Favored	86.96%	85.10%	80.22%	87.23%	83.13%
outliers	4.18%	5.30%	6.13%	3.04	4.82%
rotamers	3.92%	2.28%	4.00%	6.53	8.50%
Qmean Disco global	0.60±0.05		0.53±0.05	0.67±0.05	0.56±0.05
GMQE	0.61	0.85	0.56	0.6	0.54
Identity	93.17%	81.75%	76.59%	88.03%	83.97%
QmeanZscore	-6.69		-8.98	-4.88	-5.95
overall G factor	-0.38	-0.41	-0.45	-0.43	-0.39

G factor
* Swiss Model (SM)
